# Reactivity of the Ethenium Cation (C_2_H_5_^+^) with Ethyne (C_2_H_2_): A Combined Experimental and Theoretical Study

**DOI:** 10.3390/molecules29040810

**Published:** 2024-02-09

**Authors:** Vincent Richardson, Miroslav Polášek, Claire Romanzin, Paolo Tosi, Roland Thissen, Christian Alcaraz, Ján Žabka, Daniela Ascenzi

**Affiliations:** 1 Department of Physics, University of Trento, 38123 Trento, Italy; vincent.richardson@liverpool.ac.uk (V.R.); paolo.tosi@unitn.it (P.T.); 2 Department of Physics, University of Liverpool, Oxford Street, Liverpool L69 7ZE, UK; 3 J. Heyrovský Institute of Physical Chemistry of the Czech Academy of Sciences, Dolejšškova 2155/3, 182 23 Prague, Czech Republic; miroslav.polasek@jh-inst.cas.cz (M.P.); jan.zabka@jh-inst.cas.cz (J.Ž.); 4 Université Paris-Saclay, CNRS, Institut de Chimie Physique, UMR8000, 91405 Orsay, France; claire.romanzin@universite-paris-saclay.fr (C.R.); roland.thissen@universite-paris-saclay.fr (R.T.); christian.alcaraz@universite-paris-saclay.fr (C.A.); 5 Synchrotron Soleil, L’Orme des Merisiers, 91190 Saint-Aubin, France

**Keywords:** ion–molecule reactions, Titan, planetary atmospheres, interstellar medium, plasma, acetylene, Cassini mission

## Abstract

The gas-phase reaction between the ethyl cation (C_2_H_5_^+^) and ethyne (C_2_H_2_) is re-investigated by measuring absolute reactive cross sections (CSs) and branching ratios (BRs) as a function of collision energy, in the thermal and hyperthermal energy range, via tandem-guided ion beam mass spectrometry under single collision conditions. Dissociative photoionization of C_2_H_5_Br using tuneable VUV radiation in the range 10.5–14.0 eV is employed to generate C_2_H_5_^+^, which has also allowed us to explore the impact of increasing (vibrational) excitation on the reactivity. Reactivity experiments are complemented by theoretical calculations, at the G4 level of theory, of the relative energies and structures of the most relevant stationary points on the reactive potential energy hypersurface (PES) and by mass-analyzed ion kinetic energy (MIKE) spectrometry experiments to probe the metastable decomposition from the [C_4_H_7_]^+^ PES and elucidate the underlying reaction mechanisms. Two main product channels have been identified at a centre-of-mass collision energy of ∼0.1 eV: *(a)* C_3_H_3_^+^+CH_4_, with BR = 0.76±0.05 and *(b)* C_4_H_5_^+^+H_2_, with BR = 0.22±0.02. A third channel giving C_2_H_3_^+^ in association with C_2_H_4_ is shown to emerge at both high internal excitation of C_2_H_5_^+^ and high collision energies. From CS measurements, energy-dependent total rate constants in the range 4.3×$10^{-11}$−5.2×$10^{-10}$ ${\mathrm{cm}}^{3}\cdot$${\mathrm{molecule}}^{-1}\cdot$$s^{-1}$ have been obtained. Theoretical calculations indicate that both channels stem from a common covalently bound intermediate, CH_3_CH_2_CHCH^+^, from which barrierless and exothermic pathways exist for the production of both cyclic c−C_3_H_3_^+^ and linear H_2_CCCH^+^ isomers of the main product channel. For the minor C_4_H_5_^+^ product, two isomers are energetically accessible: the three-member cyclic isomer c−C_3_H_2_(CH_3_)^+^ and the higher energy linear structure CH_2_CHCCH_2_^+^, but their formation requires multiple isomerization steps and passages via transition states lying only 0.11 eV below the reagents’ energy, thus explaining the smaller BR. Results have implications for the modeling of hydrocarbon chemistry in the interstellar medium and the atmospheres of planets and satellites as well as in laboratory plasmas (e.g., plasma-enhanced chemical vapor deposition of carbon nanotubes and diamond-like carbon films).

## 1. Introduction

The gas-phase reactivity of ions is believed to play a significant role in the formation of complex organic molecules (COMs) in both the interstellar medium (ISM) and the upper atmospheres of planets and their satellites [1,2]. Though ions are typically less abundant, and therefore harder to detect, in the ISM than neutrals, their greater reactivity makes them more significant to the overall chemistry than their number densities indicate. Notably, the rapid reaction of charged species with neutral partners can lead to the formation of complex ions that act as important chemical intermediates since they can recombine with electrons and/or anions to produce increasingly complex neutral species under astrophysical conditions [3,4].

In our Solar System, Titan, the largest moon of Saturn, is an environment of particular interest to astrochemistry as, similar to that of the early Earth, the atmosphere of Titan is dense and dominated by N_2_, with methane (CH_4_) as the second most abundant species [5,6]. Under such conditions, a rich ion chemistry is driven by ionization and dissociative ionization of its primary components by a combination of extreme ultraviolet radiation from the Sun [7], energetic magnetospheric electrons and ions [8], and cosmic rays. The ions and radicals that are formed are then able to go on to react with surrounding neutrals, thereby leading to the formation of complex molecular species, including some with prebiotic potential [3,9,10,11,12]. Thus, “complex” ions with m/z ratios as large as 350 have been detected [9,13,14,15,16,17] by the ion neutral mass spectrometer (INMS) and Cassini plasma spectrometer–ion beam sensor (CAPS–IBS) aboard the Cassini spacecraft that made several targeted flybys of Titan [18,19].

With an estimated density of 2×$10^{2}$ ${\mathrm{cm}}^{-3}$, C_2_H_5_^+^ is thought to be the second most abundant ion in Titan’s ionosphere, where it is expected to be the major contributor to the intense *m*/*z* 29 peak observed in the mass spectra of Titan’s ionosphere measured by the Cassini INMS [14,20]. Outside Titan’s atmosphere, the presence of C_2_H_5_^+^ has been postulated on the basis of results from the Cassini Grand Finale mission [21,22,23,24], where the INMS was used to measure the composition of Saturn’s upper atmosphere and its chemical interactions with material originating in the rings [22]. Further away from our Sun, although the New Horizons mission to Pluto did not make a direct measurement of its ionosphere, C_2_H_5_^+^ has been suggested as one of the most abundant ions on the basis of the presence of hydrocarbons [25,26]. In addition, recent ALMA (Atacama large millimeter/submillimeter array) interferometric measurements have detected the presence of N-containing organics (HCN and its isomer HNC, CH_3_CN) and propyne (CH_3_CCH) in Pluto’s atmosphere [27], thus suggesting the presence of a Titan-like chemistry. C_2_H_5_^+^ has also been unambiguously identified among the diverse range of ions detected by the ROSINA high-resolution mass spectrometer employed in the Rosetta mission to comet 67P/Churyumov-Gerasimenko [28]. Though C_2_H_5_^+^ has yet to be identified in the ISM, it could feasibly be formed via the protonation of C_2_H_4_, which has been detected [29,30]. C_2_H_2_ (ethyne, also known as acetylene) is not only one of the most abundant neutral species in Titan’s ionosphere [14], but has also been detected in a range of astrochemical environments, including, but not limited to, the ionosphere of Pluto [31,32] and the ISM [33,34,35,36,37].

The reaction of C_2_H_5_^+^ with C_2_H_2_ is of particular interest as it has been shown to be one of the four most deterministic ion-neutral reactions for models of Titan’s ionosphere [38]. From previous experimental studies [39,40,41], it is well established that the main reaction products are C_3_H_3_^+^ plus CH_4_ and C_4_H_5_^+^ plus H_2_. A signature of the presence of C_3_H_3_^+^ and C_4_H_5_^+^ on Titan’s upper atmosphere has been demonstrated by the ion densities at m/z 39 and 53 obtained from the Cassini INMS data, and assigned, at least partly, to the C_3_H_3_^+^ and C_4_H_5_^+^ ions, respectively [42]. More recently, the small cyclic molecule cyclopropenylidene (c−C_3_H_2_) has been detected on Titan’s atmosphere using spectroscopic observations made with the ALMA telescope [43], and dissociative electron recombination of the cyclopropenylium cation (c−C_3_H_3_^+^) is among the mechanisms that have been proposed for the synthesis of c−C_3_H_2_. The other ionic product of the reaction, C_4_H_5_^+^, has previously been identified as a key intermediate in the formation of benzene (c−C_6_H_6_) in Titan’s ionosphere [44] and, with both C_2_H_5_^+^ and C_2_H_2_ having been identified in a range of other astrochemical environments, this reaction could well play a significant role in the formation of benzene in such environments.

In addition to the astrochemical significance of the title reaction, C_2_H_5_^+^ and C_2_H_2_ are important species in laboratory plasmas, such as those used in microwave or radiofrequency discharges in methane for plasma-enhanced chemical vapor deposition of carbon nanotubes and diamond-like carbon films [45,46], atmospheric pressure plasma reactors for non-oxidative conversion of CH_4_ into higher hydrocarbons and H_2_ [47], or for the generation of value-added fuels from CH_4_ and CO_2_ [48,49,50]. For instance, chemical kinetics models aimed at exploring the plasma chemistry operative in nanosecond pulsed discharges or dielectric barrier discharges for the partial oxidation and dry reforming of methane predict the presence of C_2_H_5_^+^ among the relevant hydrocarbon ions [51,52,53,54].

Though the reactivity of C_2_H_5_^+^ with C_2_H_2_ has been studied previously [39,40,41], our recent experimental observations [55,56] have found some inconsistencies in the relative BRs of the two main ionic products (C_3_H_3_^+^ and C_4_H_5_^+^), thereby prompting this re-evaluation of the existing literature values. This is required because accurate kinetics data, in terms of both rate constants and BRs, are essential to improve the reliability and predictive power of chemical models used both in natural and laboratory plasmas.

In this work, we present experimental BRs and reaction cross sections (CSs), as well as the results of computational calculations on the potential Energy Surface (PES) for the reaction of C_2_H_5_^+^ with C_2_H_2_. Additionally, we present data on the CSs as a function of both the collision energy and the photon energy used to generate the ions (a proxy of the internal energy) in order to probe their respective impacts, the latter being of particular relevance to the laboratory plasmas involved in the dry reforming of methane [57]. From this, we are able to identify an alternative major product at threshold from that reported in previous literature [39,40], along with BRs and reaction CSs for other minor products, including a previously unobserved channel that predominates at the higher energies relevant to conversion plasmas. These experimental observations have been complemented with computational work on the reaction PES and further experimental measurements via the technique of mass-analyzed ion kinetic energy (MIKE) spectrometry to probe the underlying dynamics behind the observed products and the implications for both the neutral fragments and the ionic isomers formed.

## 2. Experimental Results

### 2.1. Generation of C_2_H_5_^+^

The dissociative photoionization of C_2_H_5_Br has been studied extensively [58,59,60,61,62,63] with three potential pathways being relevant to the $E_{phot}$ range used as part of this work. These three pathways, along with their respective lower lying dissociation limits relative to the respective ground state C_2_H_5_Br in its zero-point vibrational level from [58], are given by Equations (1)–(3).

(1) $$\begin{array}{ccc} \begin{array}{c} C_{2}H_{5}Br+hv \end{array} & \to C_{2}{H_{5}}^{+}+{B_{r}}^{•} & +11.13\mathrm{eV} \end{array}$$

(2) $$\begin{array}{ccc} \; & \to C_{2}{H_{4}}^{+•}+{{\mathrm{HB}}_{r}}^{•} & +11.21\mathrm{eV} \end{array}$$

(3) $$\begin{array}{ccc} \; & \to C_{2}{H_{3}}^{+}+H_{2}+{\mathrm{Br}}^{•} & +13.24\mathrm{eV} \end{array}$$

**Figure 1:**
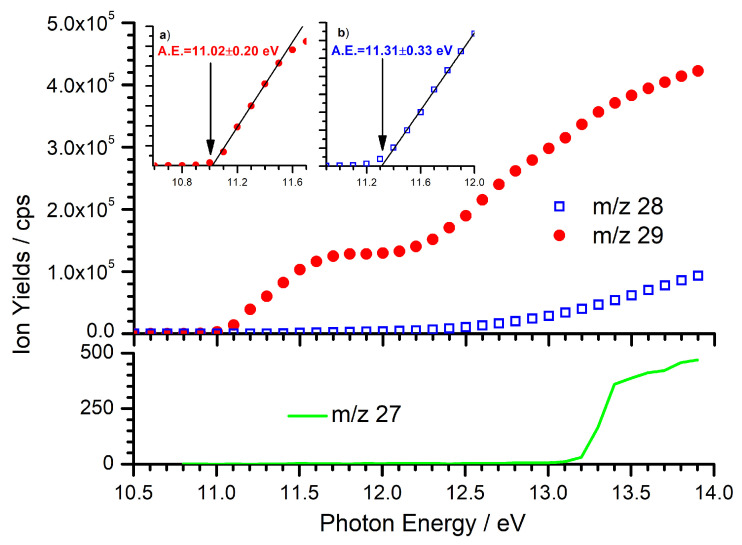
Photoionization efficiency (PIE) curves for the dissociative photoionization of C_2_H_5_Br in the 10.5–14 eV photon energy range: filled red dots are for the C_2_H_5_^+^ ion (*m*/*z* 29), open blue squares are for the C_2_H_4_^•+^ ion (*m*/*z* 28), and green line is for the C_2_H_3_^+^ ion (*m*/*z* 27). Insets (**a**) and (**b**) show enlarged plots of the threshold regions for the corresponding *m*/*z* values. The solid black lines indicate the linear extrapolations used to extract the experimental appearance energy (AE) (reported with a vertical arrow).

The fragmentation into C_2_H_5_^+^ and Br is the only channel for which experimental appearance energy (AE) values are available in the literature with the most recent values being 11.132 ± 0.008 eV [59] and 11.130 ± 0.005 eV [60]. Due to the presence of other fragments in addition to the desired C_2_H_5_^+^ ion, we conclude that we also need to consider the potential isobaric contaminant arising from ^13^CCH_4_^+•^ as this has a similar formation enthalpy to our target ion. In order to characterize the fragmentation process, we have recorded photoionization efficiency (PIE) curves (i.e., ion yields as a function of photon energy) for pathways (1)–(3), and the results are shown in Figure 1.

For the C_2_H_5_^+^ fragment ion (*m*/*z* 29) an AE of 11.02 ± 0.20 eV has been measured, which is consistent with the above-mentioned literature values [59,60] as well as older determinations [61,62]. As the intensity of the *m*/*z* 28 signal is substantially less than the predominant *m*/*z* 29 fragment, at least up to $E_{phot}∼$13 eV, we conclude that the ^13^CCH_4_^+•^ impurity should be limited to < 1% of the *m*/*z* 29 signal. However, as the *m*/*z* 28 signal increases above $E_{phot}$ = 13.0 eV, reactivity data with the C_2_H_5_^+^ ion have not been measured at $E_{phot}>$ 13.0 eV in order to limit the impact from the isobaric contaminant ^13^CCH_4_^+•^. Further consideration on the appearance energy of the *m*/*z* 28 fragment is given in the Supplementary Material.

For completeness, we have also conducted a PIE scan for the *m*/*z* 27 fragment (C_2_H_3_^+^), also shown in the bottom panel of Figure 1. Though the small signal intensity does not allow for a precise determination of the AE, for which no data exists in the literature, this pathway shows a sharp AE at ∼ 13.2 eV, in excellent agreement with the literature thermodynamic dissociation limit of 13.24 eV [58]. As a result, when $E_{phot}+E_{CM}>$ 13.2 eV, we may expect to observe a *m*/*z* 27 product corresponding to the collisional fragmentation of C_2_H_5_^+^.

The other observation of note from Figure 1 is the *m*/*z* 29 trend as a function of $E_{phot}$, which shows two approximately linear increases interspersed with a plateau between ∼11.7 and ∼12.0 eV. The large increase in the PIE signal at about 12 eV might be due to the opening of a dissociation channel in which the C_2_H_5_^+^ ion is produced in combination with a Br atom in the excited spin-orbit state Br*(^2^P_1/2_) that lies 0.457 eV above the ground state [64]. From calculations of the spin–orbit-coupled potential energy curves (PEC) for the fragmentation of the ethylbromide cation [58], it appears that the electronic ground state of C_2_H_5_Br^+^ is split into 1A′ and 1A″ states, both dissociating to ground-state C_2_H_5_^+^ plus Br, while the PEC of the electronically excited 2A′ state is dissociative along the C–Br stretch coordinate and correlates with C_2_H_5_^+^ + Br*. The calculated energetics for the two processes are 11.13 eV and 11.59 eV, respectively. This is in agreement with the He(I) photoelectron spectrum of ethyl bromide [61,65] from which the onset of the electronically excited state 2A′ is around 12 eV. In the same work, direct fragmentation from the electronically excited state of C_2_H_5_Br^+^ was observed without a prior radiationless transition to the ground electronic state. From this, we can speculate that the increase in the C_2_H_5_^+^ PIE signal around $E_{phot}$ = 12 eV might be related to the dissociative ionization from the first electronically excited state of C_2_H_5_Br^+^. Here, data as a function of $E_{CM}$ have been collected at $E_{phot}$ = 11.3, 12, and 13 eV in order to explore the different features of the C_2_H_5_^+^ PIE curve.

### 2.2. Reaction with C_2_H_2_

At low photon energies, the reaction of C_2_H_5_^+^ with C_2_H_2_ yields products at *m*/*z* 39 and 53; meanwhile, at higher $E_{phot}$, an additional product is observed at *m*/*z* 27. The most probable product channels are indicated as Equations (4)–(9), and the reaction enthalpies estimated from the literature values are summarized and compared with results from our computational calculations in Table 1.

(4) $$\begin{array}{ccc} \begin{array}{c} C_{2}{H_{5}}^{+}+C_{2}H_{2} \end{array} & \to c-C_{3}{H_{3}}^{+}+{\mathrm{CH}}_{4} & m/n\;\;39 \end{array}$$

(5) $$\begin{array}{ccc} \; & \to H_{2}{\mathrm{CCCH}}^{+}+{\mathrm{CH}}_{4} & m/n\;\;39 \end{array}$$

(6) $$\begin{array}{ccc} \; & \to c-C_{3}H_{2}{\left({\mathrm{CH}}_{3}\right)}^{+}+H_{2} & m/n\;\;53 \end{array}$$

(7) $$\begin{array}{ccc} \; & \to {\mathrm{CH}}_{2}{{\mathrm{CHCCH}}_{2}}^{+}+H_{2} & m/n\;\;53 \end{array}$$

(8) $$\begin{array}{ccc} \; & \to C_{2}{H_{3}}^{+}+C_{2}H_{4} & m/n\;\;27 \end{array}$$

(9) $$\begin{array}{ccc} \; & \to C_{2}{H_{3}}^{+}+H_{2}+C_{2}H_{2} & m/n\;\;27 \end{array}$$

Previous calculations [66] indicate that there are two isomers of C_3_H_3_^+^ that are thermodynamically feasible for this reaction, namely c−C_3_H_3_^+^ and H_2_CCCH^+^, with c−C_3_H_3_^+^ being the lowest energy isomer by 1.2–1.3 eV. The energetics of C_4_H_5_^+^ isomers have also been studied previously [67,68], with the most stable structure being the cyclic one reported in reaction (6). For completeness, as part of the computational work presented in Section 3, we have also looked for pathways leading to the higher energy isomers of [C_4_H_5_]^+^, and the only other isomer for which a submerged formation pathway has been found (at the G4 level of theory) is CH_2_CHCCH_2_^+^, reported in reaction (7). We have, therefore, limited our discussions of the *m*/*z* 53 channel to consideration of these two ions.

**Table 1:** Reaction enthalpies (Δ$H_{298}^{\circ }$) for the identified products of the reaction of C_2_H_5_^+^ with C_2_H_2_ both from ${\mathrm{literature}}^{1}$ and our ${\mathrm{calculations}}^{2,3}$. Values are given in eV.

Reaction Products	Equation	Δ$H_{298}^{\circ }$, lit. Values ^1^	ωB97X-D/cc-pVTZ ^2^	G4 ^3^
c−C_3_H_3_^+^ + CH_4_	(4)	−1.36 ± 0.10 ^4^	−1.57	−1.36
H_2_CCCH^+^ + CH_4_	(5)	−0.16 ± 0.10 ^4^	−0.25	−0.19
c−C_3_H_2_(CH_3_)^+^ + H_2_	(6)	−1.68 ± 0.10 ^5^	−1.77	−1.57
CH_2_CHCCH_2_^+^ + H_2_	(7)	−1.01± 0.10 ^5^	-	−0.92
C_2_H_3_^+^ + C_2_H_4_	(8)	+0.39 ± 0.08 ^6^	+0.37	+0.36
C_2_H_3_^+^ + H_2_ + C_2_H_2_	(9)	+2.21 ± 0.08 ^6^	+2.29	+2.09

^1^ All values in this column have been estimated using the following formation enthalpies: $\Delta_{f}$$H_{298}^{\circ }$(C_2_H_5_^+^) = 9.36±0.03 eV [69] and $\Delta_{f}$$H_{298}^{\circ }$(C_2_H_2_) = 2.35±0.01 eV [70]. ^2^ Present work: calculations at the ωB97X-D/cc-pVTZ level of theory have been performed using Gaussian 16 [71]. ^3^ Present work: calculations at the G4 level have been performed using Gaussian 16 [71]. ^4^ Formation enthalpy for CH_4_ is from NIST [70], while the values for c−C_3_H_3_^+^ and H_2_CCCH^+^ are from Ref. [69]. ^5^ Formation enthalpies of c−C_3_H_2_(CH_3_)^+^ and CH_2_CHCCH_2_^+^ are from calculations at the MP4SDTQ/6-311++G(2df,p) level of theory from Cunje et al. [67]. ^6^ Formation enthalpy for C_2_H_4_ is from NIST [70], while that of C_2_H_3_^+^ is from Lago et al. [72].

The rate constant and product distributions (BRs) of the reaction between C_2_H_5_^+^ and C_2_H_2_ have been measured previously using an ion cyclotron resonance (ICR) mass spectrometer, where C_2_H_5_^+^ ions were generated using electron ionization of C_2_H_5_Cl and C_2_H_6_ with a nominal electron energy of 16 eV [39]. The measured total rate constant was 1.9×$10^{-10}$ ${\mathrm{cm}}^{3}\cdot$${\mathrm{molecule}}^{-1}\cdot$$s^{-1}$ (somewhat larger than an older value of 1.0×$10^{-10}$ ${\mathrm{cm}}^{3}\cdot$${\mathrm{molecule}}^{-1}\cdot$$s^{-1}$ reported in [73]), and the two products at *m*/*z* 39 and 53 were observed with BRs of 0.36 and 0.64, respectively. A comparison of the BRs collected as part of this work (at $E_{phot}$ = 11.3 eV, $E_{CM}∼0.1$ eV) with those from Kim et al. [39] is made in Table 2. It should be noted that, while previous experiments found the reaction channel [C_4_H_5_]^+^ plus H_2_ to be the dominant one with a BR = 0.64, present results show that the main channel is the production of [C_3_H_3_]^+^ in association with a CH_4_ molecule, with a BR = 0.76±0.05. A more detailed consideration on this discrepancy is given in Section 4.2 and Section 4.3.

The data presented here therefore not only involve the identification of a new product channel at *m*/*z* 27 (C_2_H_3_^+^) that is particularly relevant for higher energy plasmas, but also a significant update to the BRs at low internal energies. This latter point, in particular, could potentially lead to a marked change in the predicted abundances of higher mass ions in Titan’s ionosphere [38], with further modeling work required for a quantitative assessment to be made.

**Table 2:** Branching ratios (BRs) for the products for the reaction of C_2_H_5_^+^ with C_2_H_2_ measured at $E_{phot}$ = 11.3 eV and $E_{CM}∼0.1$ eV.

Ionic Reaction Products	Product Mass (*m*/*z*)	Equations	BR, This Work	Literature BR ^1^
C_2_H_3_^+^	27	(8)/(9)	0.02 ± 0.02	0.00
C_3_H_3_^+^	39	(4)/(5)	0.76 ± 0.05	0.36
C_4_H_5_^+^	53	(6)/(7)	0.22 ± 0.02	0.64

^1^ Kim et al. [39].

Table 3 reports the energy-dependent total rate constants $k_{tot}\left(E_{ave}\right)$ obtained from the CSs measured as a function of the collision energy at $E_{phot}=11.3$ eV (to be presented in Section 2.2.2) The energy-dependent total rate constants have been estimated using the expression:

(10) $$k_{tot}\left(E_{ave}\right)=<v>\cdot \sigma_{tot}$$

where $\sigma_{tot}$ is the total reaction CS (summed over all the product channels), and <v> is the average relative velocity that can be estimated from the collision energy $E_{CM}$ (see [74,75] for a more detailed treatment). It should be noted that the rates obtained at the hyperthermal velocities probed in our experiment are comparable with the value 1.9×$10^{-10}$ ${\mathrm{cm}}^{3}\cdot$${\mathrm{molecule}}^{-1}\cdot$$s^{-1}$ determined via ICR mass spectrometry [39] and are smaller than the rate estimated from a simple Langevin capture model ($1.18\times 10^{-9}$ ${\mathrm{cm}}^{3}\cdot$${\mathrm{molecule}}^{-1}\cdot$$s^{-1}$, see last column of Table 3).

The significant under-observation with respect to the Langevin rate is unsurprising, as the model assumes that if the reactants have enough energy to surmount the centrifugal barrier, the reaction forms from the resultant ion–molecule complex with unit probability, with no accounting for any dissociation back to the reactants. Not only has this assumption been shown to be false in a number of previous studies [55,56,76], but the limitations of capture theories in providing quantitative assessments of reaction rates, especially for complex systems such as in the current study, have been explored extensively in literature [77].

#### 2.2.1. Cross Sections as a Function of the Photon Energy

**Figure 2:**
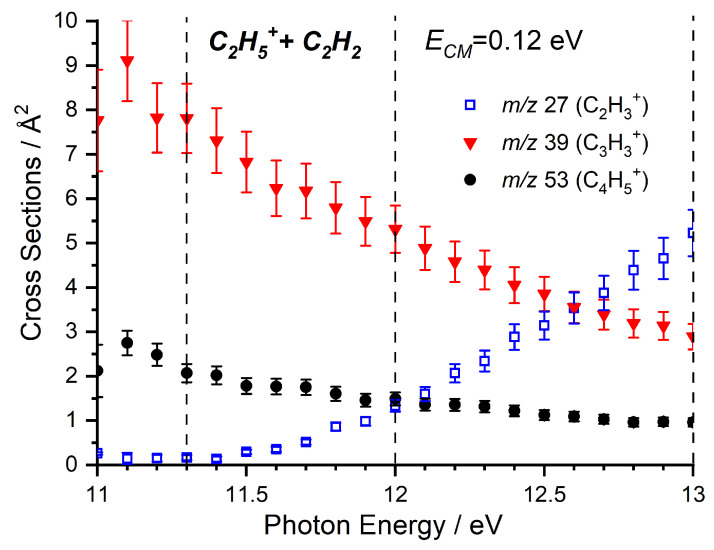
Cross sections (CSs) as a function of $E_{phot}$ for the *m*/*z* 27, 39, and 53 products of the reaction of C_2_H_5_^+^ with C_2_H_2_ at $E_{CM}$ = 0.12 eV. Dashed lines represent the photon energies at which CSs have been measured as a function of the collision energy for the various product channels.

Absolute CSs as a function of $E_{phot}$ have been collected at low collision energy for the *m*/*z* 27 (C_2_H_3_^+^), 39 (C_3_H_3_^+^) and 53 (C_4_H_5_^+^) products, with results presented in Figure 2. The two products that are most abundant at low $E_{phot}$, namely *m*/*z* 39 and 53, show a marked decrease in the CS with increasing $E_{phot}$, while the *m*/*z* 27 product shows an increase above its AE of ∼11.4 eV. Importantly, we note that the *m*/*z* 39 and 53 signals decrease below the AE of the *m*/*z* 27 product and so, while we expected the competition between the channels to contribute to the decrease as $E_{phot}$ is increased, we must also consider what leads to the initial decrease in CS for the *m*/*z* 39 and 53 products.

**Figure 3:**
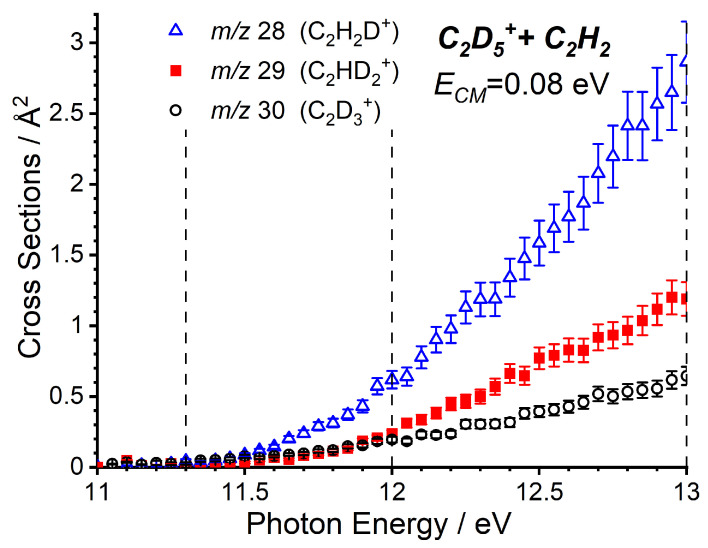
Cross sections (CSs) as a function of $E_{phot}$ for the *m*/*z* 28, 29, and 30 products of the reaction of C_2_D_5_^+^ with C_2_H_2_ at $E_{CM}$ = 0.08 eV. Dashed lines represent the photon energies at which CSs have been measured as a function of the collision energy for the various product channels.

As the *m*/*z* 27 product (C_2_H_3_^+^) can correspond with either the endothermic proton transfer, represented by reaction (8), or the collision-induced dissociation (CID) of the parent ion, reaction (9), further data are required to be able to elucidate the relative contributions of these two channels. To do this, we have also performed a more limited study on the reactivity of C_2_D_5_^+^ with C_2_H_2_, where the direct proton (deuteron in this case) transfer product C_2_H_2_D^+^ will appear at *m*/*z* 28, while the CID of the parent ion to give C_2_D_3_^+^ will be shifted to *m*/*z* 30, with complex- and adduct-mediated processes leading to a level of H/D scrambling to give products at *m*/*z* 28, 29, and 30. The trends of these three products as a function of the photon energy are shown in Figure 3, where a clear preference is shown for the *m*/*z* 28 product, indicating that the observed signal at low collision energies predominately corresponds to the proton transfer.

The observation of minor products at *m*/*z* 29 and 30 at low collision energies, as well as the relative intensities of the three products with C_2_H_5_^+^ under different conditions are discussed in more detail in Section 2.2.2.

#### 2.2.2. Cross Sections as a Function of the Collision Energy

**Figure 4:**
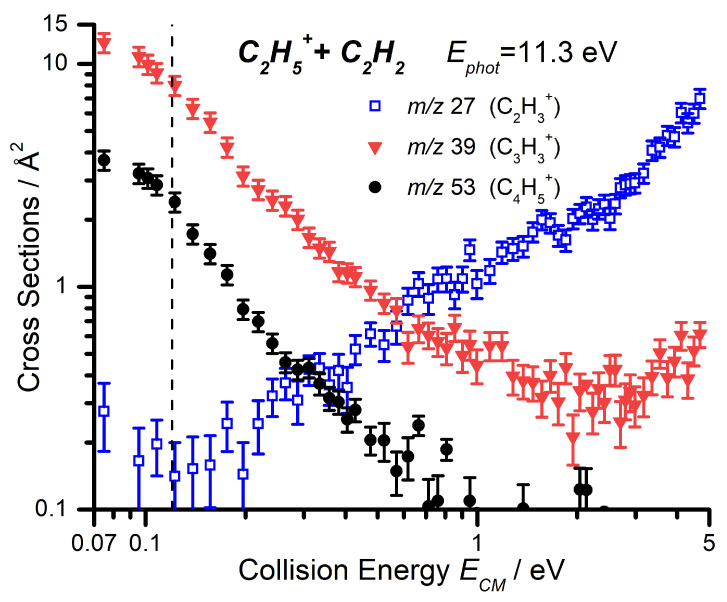
Cross sections as a function of $E_{CM}$ (in log–log scale) for the *m*/*z* 27, 39, and 53 products of the reaction of C_2_H_5_^+^ with C_2_H_2_ at $E_{phot}$ = 11.3 eV. The dashed line represents the collision energy at which the corresponding scan as a function of the photon energy (Figure 2) was conducted.

**Figure 5:**
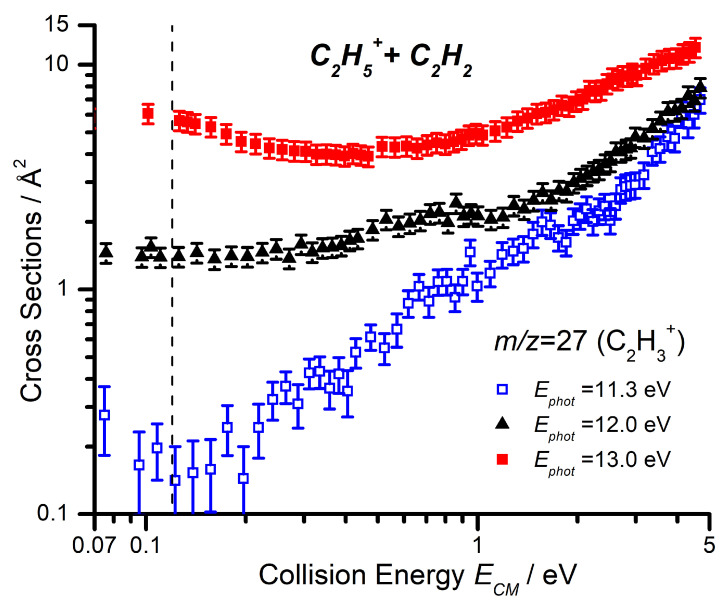
Cross sections as a function of $E_{CM}$ (in log–log scale) for the *m*/*z* 27 product (C_2_H_3_^+^) of the reaction of C_2_H_5_^+^ with C_2_H_2_, at $E_{phot}$ = 11.3, 12.0, and 13.0 eV. The dashed line represents the collision energy at which the corresponding scan as a function of the photon energy (Figure 2) was conducted.

For each of the three products, data as a function of $E_{CM}$ have been collected at $E_{phot}$ = 11.3, 12.0, and 13.0 eV, in order to explore the combined effects of $E_{phot}$ and $E_{CM}$ on the reaction. Here, we present both the CS of the three channels as a function of the collision energy at the lowest photon energy of 11.3 eV (Figure 4) and a comparison of the CS as a function of the collision energy for the *m*/*z* 27 product at the three different photon energies (Figure 5).

The formation of the *m*/*z* 39 product ([C_3_H_3_]^+^) is the major pathway at low collision energies (as observed in Figure 2), but its CS decreases with increasing collision energy, consistent with a pathway proceeding via formation of an adduct, with a subsequent increase with increasing collision energy that emerges at $E_{cm}>$ 2 eV. This is a general trend since the collision energy shortens the precursor lifetime and drastically reduces the reaction efficiency. The inhibition of the low collision energy feature by increasing photon energy has already been shown in Figure 2, while the high collision energy feature is approximately independent of the photon energy, and so, a comparison of the collision energy dependence of this channel at different photon energies is limited to Figure S1 in the Supplementary Material. However, we briefly note here that, as can be seen through comparison with Figure S2 in the Supplementary Material, the high-collision energy rise in the *m*/*z* 39 signal is matched by a corresponding decrease in *m*/*z* 53 signal, indicating that this is a real effect and not an instrumental artefact.

The *m*/*z* 53 product ([C_4_H_5_]^+^) exhibits a similar decrease with increasing collision energy to the *m*/*z* 39 product, again consistent with a complex-mediated pathway due to the reduction of precursor lifetime with increasing collision energy, as outlined in the previous paragraph. However, unlike the *m*/*z* 39 product, this is not accompanied by an increase in CS at high collision energies. As with the *m*/*z* 39 product, the inhibition of the low collision energy feature by increasing photon energy has already been shown in Figure 2, and so, a comparison of the collision energy dependence of this channel at different photon energies is limited to Figure S2 in the Supplementary Material.

The *m*/*z* 27 product (C_2_H_3_^+^) shows a significant increase in CS with increasing collision energy, starting from around 0.2–0.3 eV. When we compare this trend at different photon energies (Figure 5), we note that not only does the measured CS increase with increasing photon energy over the entire collision energy range, but that this increase is especially pronounced at low collision energies. It is important to note that the internal energy variations of the C_2_H_5_^+^ with changes in the photon energy are not easily quantifiable since, in the dissociative ionization process, a fraction of the photon energy is taken by both the ejected electron and the Br atom fragment. However, the observed trends with photon energy point to an increase in the amount of internal energy available to the C_2_H_5_^+^ reagent ion.

**Figure 6:**
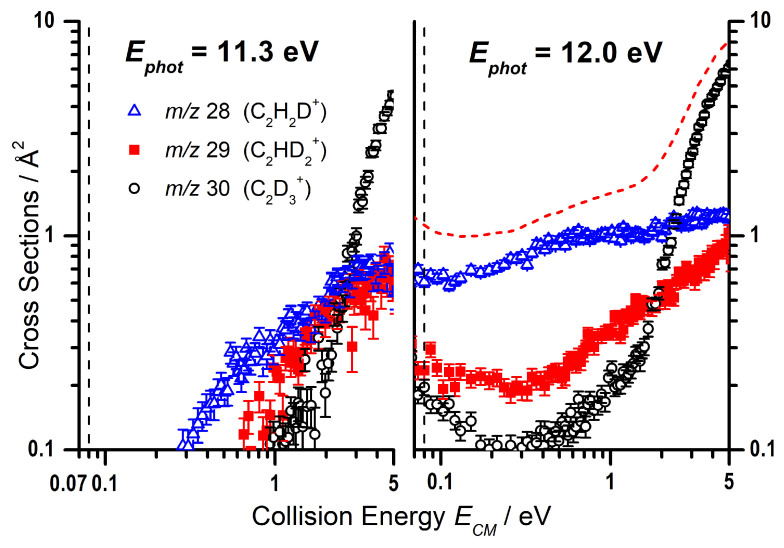
Cross sections (CSs) as a function of $E_{CM}$ (in log–log scale) for the *m*/*z* 28, 29, and 30 products of the reaction of C_2_D_5_^+^ with C_2_H_2_ at $E_{phot}$ = 11.3 eV (left panel) and 12.0 eV (right panel). The red dashed line is a smoothed sum of the CSs for the three channels. The black dashed lines represent the collision energy at which the corresponding scans as a function of the photon energy (Figure 3) were conducted.

As with the photon energy dependence, we have collected data for the reaction of C_2_D_5_^+^ with C_2_H_2_ as a function of the collision energy for the *m*/*z* 28, 29, and 30 products, at both $E_{phot}$ = 11.3 and 12.0 eV, with the results shown in Figure 6. As already mentioned, the deuteron transfer product (C_2_H_2_D^+^) will appear at *m*/*z* 28, while the CID of the parent ion to give C_2_D_3_^+^ will be shifted to *m*/*z* 30. From Figure 6 it can be noted that, at $E_{phot}$ = 11.3 eV, the *m*/*z* 30 product is only observed at collision energies greater than ∼1 eV, while the deuteron transfer at *m*/*z* 28 shows an appearance energy of about 0.3 eV that is not so dissimilar from the expected reaction endothermicity of Equation (8) (See Table 1). It is therefore possible to assign the observed *m*/*z* 27 CS in Figure 5 below a collision energy of ∼1 eV, at $E_{phot}$ = 11.3 eV, to the endothermic proton transfer.

However, while a direct deuteron transfer mechanism can explain the observed *m*/*z* 28 signal, the *m*/*z* 29 product in Figure 6 can be rationalized only by assuming a contribution from H/D scrambling from a complex mediated mechanism, that would give C_2_HD_2_^+^ in addition to the other two channels:

(11) $$\begin{array}{ccc} \begin{array}{c} C_{2}{D_{5}}^{+}+C_{2}H_{2}\to {\left[C_{4}D_{5}H_{2}\right]}^{+} \end{array} & \to C_{2}H_{2}D^{+}+C_{2}D_{4} & m/n\;28 \end{array}$$

(12) $$\begin{array}{ccc} \; & \to C_{2}{{\mathrm{HD}}_{2}}^{+}+C_{2}{HD}_{3} & m/n\;29 \end{array}$$

(13) $$\begin{array}{ccc} \; & \to C_{2}{D_{3}}^{+}+C_{2}H_{2}D_{2} & m/n\;30 \end{array}$$

By focusing only on the data at low collision energies (i.e., below ∼1 eV), the larger CS values observed at the larger photon energy $E_{phot}$ = 12.0 eV (right panel of Figure 6) compared to $E_{phot}$ = 11.3 eV (left panel of Figure 6) for the *m*/*z* 30, 29, and 28 products can therefore be rationalized as due to the direct and complex mediated deuteron transfer reactions (11)–(13), the probability of which increases due to the larger amount of internal energy available to the reacting cation. The red dashed line is a smoothed sum of the CSs for the three channels, to be compared with the collision energy trend for the *m*/*z* 27 product of Figure 5 measured at the same $E_{phot}$ = 12.0 eV: the similarity in absolute value and trend of the CSs is an indication that the interpretation in terms of scrambling in complex mediated deuteron transfer is feasible.

### 2.3. MIKE Spectra

**Figure 7:**
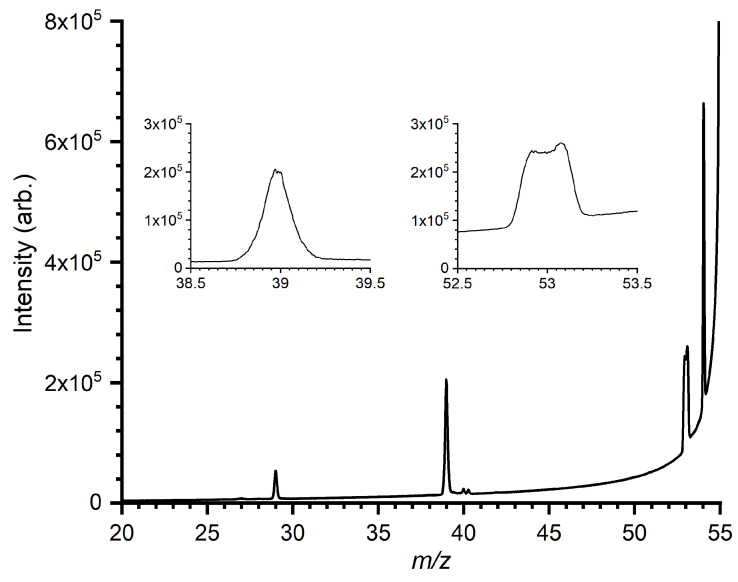
Mass-analyzed ion kinetic energy (MIKE) spectrum of the *m*/*z* 55 ion formed via dissociative ionization of 2-methylpropene. Insets show shapes of the *m*/*z* 39 and 53 peaks, while the peak at *m*/*z* 54 is an artefact arising from leaking of *m*/*z* 56 ions into the precursor ion signal.

In order to probe the metastable decomposition from the [C_4_H_7_]^+^ PES, MIKE spectra have been recorded for CH_2_C(CH_3_)CH_2_^+^ ions generated via dissociative ionization of 2-methylpropene (CH_2_C(CH_3_)_2_), with the results shown in Figure 7. Full KER distributions of the *m*/*z* 39 and 53 products have been determined from the metastable peak shapes using the META algorithm described in [78].

The peak at *m*/*z* 53, corresponding to the loss of H_2_, has a dish-topped shape characteristic of a process involving a large kinetic energy release (KER). Contrastingly, the *m*/*z* 39 peak, corresponding to the loss of CH_4_, has a markedly sharper profile, characteristic of a process that not only involves a lower KER but also a narrower KER distribution. The obtained KER distributions of the *m*/*z* 39 and 53, products are given in Figures S3 and S4, respectively, in the Supplementary Material. The peak at *m*/*z* 29 corresponds to the ejection of C_2_H_5_^+^, which is the entrance channel in the title reaction.

An equivalent MIKE spectrum of [C_4_D_7_]^+^ has also been recorded, in this case starting from d_8_-2-methylpropene (CD_2_C(CD_3_)_2_), where, despite the shifting of peak masses due to deuteration, very similar peak shapes are observed. This is shown in Figure S5 in the Supplementary Material, with the corresponding KER distributions for the *m*/*z* 42 and 58 fragments given by Figures S6 and S7, respectively. A further discussion of these results is given in Section 4.

## 3. Computational Results

In order to rationalize the observed products and trends as a function of both $E_{phot}$ and $E_{CM}$, the PES of the reaction has been explored through computational calculations, as described in Section 5.2. The PES was initially mapped at the ωB97X-D/cc-pVTZ level of theory, but a comparison of the relative energies obtained with those calculated from literature formation enthalpies (as shown in Table 1) indicated that a higher level of theory is required. The PES shown here is, therefore, that calculated at the G4 level of theory.

Results of the calculations are in terms of the stationary points along the reaction pathways and are shown in Figure 8 and Figure 9, where the zero energy is given by the sum of the energies of the reactants, and the relative energies for species (reported in parentheses in the following) are in eV. Structural diagrams and atomic coordinates of the intermediates and transition states are given in Figures S9–S45 and Tables S4–S40 of the Supplementary Material. An overview of the reaction pathways and rearrangements of the intermediates is summarized in Scheme 1.

For all products at low $E_{CM}$, the reaction proceeds via the entrance channel ion-induced dipole complex **W1** (−0.44), from which the covalently-bound adduct CH_3_CH_2_CHCH^+^ (−1.16), hereafter referred to as **M1**, can form via the transition state **W1-M1** (−0.28). Though endothermic, the *m*/*z* 27 product C_2_H_3_^+^ can form in combination with C_2_H_4_ (0.36) either directly from the reagents or via **W1**. In both cases, this proceeds without a barrier.

As already mentioned and shown in Table 1, for the *m*/*z* 39 product, both the cyclic (c−C_3_H_3_^+^) and linear (H_2_CCCH^+^) isomers are energetically allowed, with the former being more stable by ∼1.2 eV, see reactions (4) and (5). Similarly, for the *m*/*z* 53 product, both the cyclic (c−CHCHC(CH_3_^+^) and linear (CH_2_CHCCH_2_^+^) isomers are energetically allowed, with the former being more stable by ∼0.6–0.7 eV, see reactions (6) and (7).

From **M1**, the lower energy c−C_3_H_3_^+^ isomer of [C_3_H_3_]^+^ can form following a simultaneous [1,3] cyclization and [1,2] H-shift to give **W2** (−1.47), an ion-induced dipole complex of c−C_3_H_3_^+^ and CH_4_, via **M1-W2** (−1.06). **W2** is then able to fragment into the separated products (−1.36) without a barrier. IRC calculations on the transformation from **M1** into products have been performed and are presented in the Supplementary Material (as animated *gif* file *TS M1-W2.gif*).

The lower energy c−C_3_H_2_(CH_3_)^+^ isomer of [C_4_H_5_]^+^ can form from **M1** via an initial [1,3] H-shift to give the *anti-* conformer of CH_3_CHCHCH_2_^+^ (−2.77), hereafter referred to as **M2**, via **M1-2** (−0.96). **M2** can then undergo a [1,2] H-shift to give CH_3_CHCCH_3_^+^ (−2.05), hereafter referred to as **M3**, via **M2-3** (−1.27). **M3** can then undergo a simultaneous [1,3] cyclization and H_2_-ejection via **M3-W3** (−0.11) to give **W3** (−1.58), an ion-induced dipole complex of c−C_3_H_2_(CH_3_)^+^ and H_2_ that can fragment into the separated products (−1.57) without a barrier. IRC calculations on the transformation from **M3** into products have been performed and are presented in the Supplementary Material (as animated gif file *TS M3-W3.gif*): the methyl group migrates to the carbene C atom, thus closing the three-member ring, and, as a consequence of such closure, two H atoms are eliminated from the migrating methyl group in the form of an H_2_ molecule.

The higher energy *m*/*z* 39 and 53 isomers, H_2_CCCH^+^ and CH_2_CHCCH_2_^+^, can both form from **M2** via an initial interconversion into the higher energy *syn-*conformer of CH_3_CHCHCH_2_^+^ (−2.63), hereafter referred to as **M4**, via **M2-4** (−1.84). It should be noted that **M4** can also be accessed from **M3** via a [1,2] H-shift over **M3-4** (−1.29). **M4** can then undergo a [1,2] H-shift between the two central C atoms to give CH_3_CH_2_CCH_2_^+^ (-1.84), hereafter referred to as **M5**, via **M4-5** (−1.63). **M5** can then undergo a simultaneous C-C cleavage and [1,2] H−shift between the CH_2_ and CH_3_ moieties via **M5-W4** (−0.26) to give **W4** (−0.31), an ion-induced dipole complex of CH_2_CCH^+^ and CH_4_. **W4** can then fragment into the separated products (−0.19) without a barrier. IRC calculations on the transformation from **M5** into such products have been performed and are presented in the Supplementary Material (as animated *gif* file *TS M5-W4.gif*).

Alternatively, **M5** can undergo a [1,2] H_2_-ejection via **M5-W5** (−0.11) to give **W5** (−0.93), a weakly bound adduct of CH_2_CHCCH_2_^+^ and H_2_, that can again fragment into the separated products (−0.92) without a barrier. IRC calculations on the transformation from **M5** into such products have been performed and are presented in the Supplementary Material (as animated *gif* file *TS M5-W5.gif*).

**Figure 8:**
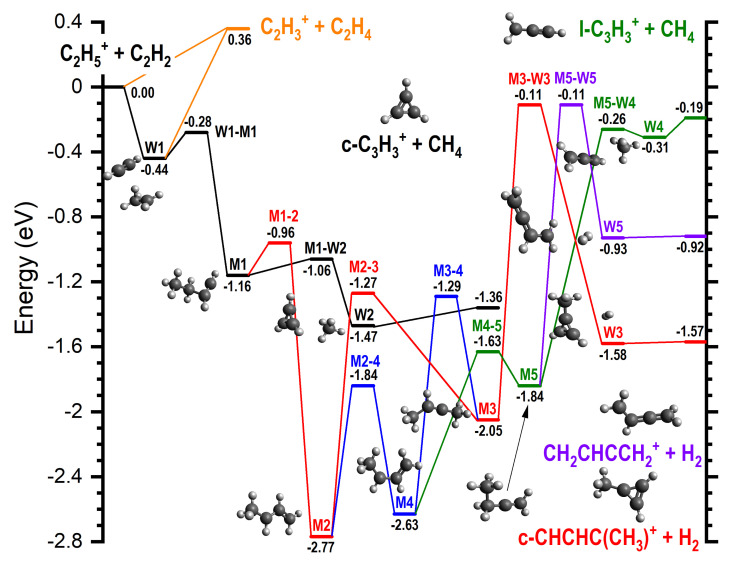
The potential energy surface for the reaction of C_2_H_5_^+^ with C_2_H_2_: the most relevant reaction pathways, energetically accessible in the experiments and including intermediates and transition states (TSs), leading to the formation of *m*/*z* 27, 39, and 53 products are reported. Energies are computed, as detailed in the text, at the G4 level of theory. The zero of the energy scale is given by the sum of the reagent energies. For clarity reasons, only some of the intermediates’ structures are reported. All the structures for intermediates and TSs are available in the Supplementary Material.

Finally, when sufficient internal energy is available, the endothermic proton transfer channel to give C_2_H_3_^+^ (*m*/*z* 27) and C_2_H_4_ (+0.36) can proceed either directly from the reactants or from the initial **W1** complex. All of these reaction pathways are shown graphically in Figure 8. As discussed earlier, at higher collision energies, there is also the possibility for collision-induced dissociation (CID) of the parent ion into C_2_H_3_^+^ and H_2_, with the calculated reaction enthalpy for this process (Table 1) showing a good level of agreement, at both levels of theory, with the literature value.

**Figure 9:**
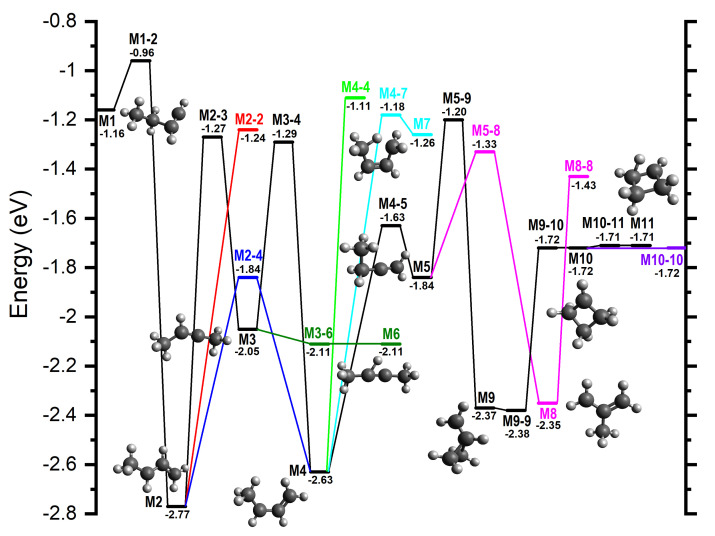
Structure and energies of the intermediates and transition states (TSs) relevant to the inter-conversion of [C_4_H_7_]^+^ adducts formed by the reaction of C_2_H_5_^+^ with C_2_H_2_. Energies are computed, as detailed in the text, at the G4 level of theory. The zero of the energy scale is given by the sum of the reagent energies. For clarity reasons, only some of the intermediate structures are reported. All the structures, for intermediates and TSs are available in the Supplementary Material.

In addition to calculations on the pathways leading to products, we have also probed the [C_4_H_7_]^+^ PES more widely in order to explore minima and transition states relevant to H/D scrambling, with the results shown graphically in Figure 9, where the zero energy is again given by the sum of the energies of the reactants.

Starting from **M2**, the first rearrangement to note is a [1,4] H-shift along the C atom chain that produces a mirrored **M2** intermediate via **M2-2** (−1.24). A similar [1,4] H-shift is also possible from **M4** via **M4-4** (−1.11). From **M3**, there is the potential for the CH hydrogen to adopt a bridged position between the two central C atoms via **M3-6** (−2.11) to give **M6** (−2.11). From **M4**, a partial delocalization of one of the methyl hydrogens between the two terminal C atoms to give the strained **M7** structure (−1.26) is possible via **M4-7** (−1.18).

Two further interconversion pathways proceed from **M5**, with the lowest energy being a [1,2] CH_3_-shift to give CH_2_C(CH_3_)CH_2_^+^ (−2.35), hereafter referred to as **M8**, via **M5-8** (−1.33). The resultant structure is then able to isomerize into itself via a [1,3] H-shift over **M8-8** (−1.43). **M5** is also able to undergo a simultaneous cyclization and [1,3] H-shift via **M5-9** (−1.20) to give c−CH_2_CH_2_CH(CH_2_)^+^ (−2.37), hereafter referred to as **M9**.

From **M9** two four-membered ring structures, **M10** (−1.72) and **M11** (−1.71), can be formed. The $C_{s}$ symmetric **M11** is, in fact, a π-protonated cyclobutene, which easily isomerizes by H-migration via **M10-11** (−1.71) to the $C_{s}$-symmetric cyclobutylium ion **M10**. The ring in **M10** is slightly puckered with a C(H)-C(H_2_)-C(H_2_)-C(H_2_) dihedral angle of 6.8°. A slight increase of this angle while maintaining $C_{s}$ symmetry leads to **M9-10** (−1.72) from which the system goes downhill, reaches the valley–ridge inflection point, and proceeds further to another TS. This type of PES describes a reaction mechanism that has been referred to as a two-step-no-intermediate [79]. The latter TS is also a barrier for isomerization of **M9** into itself, i.e., **M9-9** (−2.38). From **M10**, planarizing the ring leads to a $C_{2v}$ symmetric structure representing the ring inversion transition state **M10-10** (−1.72).

Though the scrambling is discussed in more detail in Section 4 and in Section 5 of the Supplementary Material, we draw attention to the fact that almost all the additional minima identified in Figure 9 are symmetric, allowing for efficient exchange of H atoms.

## 4. Discussion

From the calculations presented in Section 3, it is possible to rationalize the experimental CSs and their trends with collision energy for the various products.

### 4.1. Product at *m*/*z* 27: C_2_H_3_^+^

As already mentioned, two different pathways are possible for the formation of C_2_H_3_^+^ ions (*m*/*z* 27): the endothermic proton transfer channel (8) and the CID of the reagent ion (9). Through the CSs measured for the reactivity of C_2_D_5_^+^ with C_2_H_2_ (see Figure 3 and Figure 6), we have been able to assess that both pathways are operative in different collision energy regimes. In particular, the production of C_2_H_3_^+^ at high collision energies (larger than ∼1 eV) is assigned to the CID of the parent ion, while the product observed at lower energy is the result of the endothermic proton transfer mediated by the electrostatically bound ion–molecule complex **W1**.

### 4.2. Products at m/z 39 ([C_3_H_3_]^+^) and m/z 53 ([C_4_H_5_]^+^)

For the [C_3_H_3_]^+^ (*m*/*z* 39) and [C_4_H_5_]^+^ (*m*/*z* 53) products, calculations indicate that they both proceed via the **M1** covalently-bound intermediate formed from **W1**. The decrease in CS with increasing $E_{phot}$ for both channels (see Figure 2) can be rationalized by a combination of two factors: (i) the increased competition due to the emergence of the endothermic channels (8) and (9) giving C_2_H_3_^+^ (*m*/*z* 27) and (ii) the inhibition of adduct formation when the internal energy content of the reagent ion increases.

#### 4.2.1. Rationalization of *m*/*z* 39 and 53 Branching Ratios

To rationalize the BRs for the *m*/*z* 39 and 53 products, several aspects should be considered. From a thermochemical point of view, the calculated energies at the G4 level (see Table 1) indicate that the production of the lowest energy isomer c−C_3_H_2_(CH_3_)^+^ (*m*/*z* 53) plus H_2_, reaction (6), with Δ$H_{298}^{\circ }$ = −1.57 eV, is more exothermic than the CH_4_ elimination to give either c−C_3_H_3_^+^ via reaction (4) (by 0.21 eV), or l−C_3_H_3_^+^ via reaction (5) (by 1.38 eV). On the other hand, the production of the higher energy isomer CH_2_CHCCH_2_^+^ (*m*/*z* 53) plus H_2_, reaction (7) with an exothermicity Δ$H_{298}^{\circ }$ = −0.92 eV, is thermodynamically favoured over reaction (5) but not over reaction (4).

From a kinetic perspective, we must consider both the relative heights of the transition states and the number of rearrangements required prior to fragmentation. As all four relevant product channels stem from the initial adduct **M1**, we only need to consider the kinetics starting from this point.

For the *m*/*z* 39 products, the formation of c−C_3_H_3_^+^ proceeds via a single interconversion over **M1-W2**, which has a relative energy of −1.06 eV. In contrast, the formation of CH_2_CCH^+^ proceeds via four interconversions over **M1-2** (−0.96), **M2-4** (−1.84), **M4-5** (−1.63), and **M5-W4** (−0.26). The formation of the cyclic isomer is therefore not only more exothermic than the formation of the linear isomer, but is also significantly more favored kinetically.

For the *m*/*z* 53 products, both isomers proceed following the interconversion to **M2** via **M1-2**, and so the relative kinetic feasibility can be considered from this point. For the c−C_3_H_2_(CH_3_)^+^ isomer, the formation can then proceed following an isomerization to **M3** via **M2-3** (−1.27) and rearrangement via **M3-W3** (−0.11). For the CH_2_CHCCH_2_^+^ isomer, however, formation requires three successive rearrangements via **M2-4**, **M4-5**, and **M5-W5** (−0.11). While the maximum barrier heights for these two processes are the same, the much greater length of the formation pathway for the CH_2_CHCCH_2_^+^ isomer means that this pathway is much less favorable from a kinetic perspective.

#### 4.2.2. Comparison of Computed Energy Pathways with MIKE Spectra

These conclusions are supported by the MIKE spectra collected as part of this work. Starting from **M8** (see Figure 9), the [C_4_H_7_]^+^ ions formed via the dissociative ionization of the 2-methylpropane can then isomerize to give **M5**, from which all five fragmentation pathways (including that to give C_2_H_5_^+^ plus C_2_H_2_) are then accessible (see Figure 8). From the broad shape of the *m*/*z* 53 peak (Figure 7), we are able to conclude that the [C_4_H_5_]^+^ fragment ions are formed with a large KER, consistent with fragmentation products that are much lower in energy than the adjacent transition state. This is consistent with the formation of the c−C_3_H_2_(CH_3_)^+^ isomer, where the energy of the products (−1.57) is significantly lower than the transition state, **M3-W3** (−0.11), leading to the fragmentation. Should the major formation pathway be that to give the linear CH_2_CHCCH_2_^+^, we would instead observe a narrow distribution consistent with the higher energy of the products (−0.92) relative to the barrier to fragmentation via **M5-W5** (−0.11).

By contrast, for the *m*/*z* 39 peak, the sharp distribution (Figure 7) is consistent with a fragmentation pathway where the energy of the products lies only slightly lower than the barrier to fragmentation. Again comparing this to the PES, we note that it is consistent with the lower-energy pathway to form c−C_3_H_3_^+^, where the products (−1.36) are only 0.3 eV lower than the barrier to fragmentation (**M1-W2**, −1.06), with the formation of the CH_2_CCH^+^ isomer involving products which are higher in energy than the barrier to fragmentation.

The total KER distributions obtained from the *m*/*z* 39 and 53 peaks of the MIKE spectra are reported in Figures S3 and S4 of the Supplementary Material, respectively, and show a clear difference in the distributions for the two channels. For the H_2_ elimination channel, there is a tail that extends up to ∼ 1.1 eV, while that for CH_4_ elimination is much smaller, extending only up to ∼ 0.30 eV. The KER distributions for the equivalent *m*/*z* 42 (C_3_D_3_^+^) and 57 (C_4_D_5_^+^) peaks in the MIKE spectrum of the deuterated analog (see Figure S5 of the Supplementary Material) are reported in Figures S6 and S7 of the Supplementary Material, with the high level of agreement between the hydrogenated and deuterated samples indicating that there is little to no contribution to the fragmentation from tunneling. While a comparison of the KER distributions with the calculated energies indicates that a fraction of the available energy is transferred into internal excitation of the fragments, a quantification of the effect is beyond the scope of the work.

Given the observed peak profiles from the MIKE spectra and the calculated PES, we are therefore able to conclude that the *m*/*z* 39 and 53 products correspond to c−C_3_H_3_^+^ and c−C_3_H_2_(CH_3_)^+^, respectively.

When focusing on the kinetics of the formation of c−C_3_H_3_^+^ and c−C_3_H_2_(CH_3_)^+^, i.e., the predominant isomer for the two main mass channels, we note that the formation of c−C_3_H_3_^+^ (*m*/*z* 39) not only involves a much lower reaction barrier than the formation of c−C_3_H_2_(CH_3_)^+^ (*m*/*z* 53), but also requires one fewer rearrangement prior to fragmentation. Therefore, while the formation of the *m*/*z* 53 (c−C_3_H_2_(CH_3_)^+^) product is the most exothermic pathway, the calculated PES is consistent with the experimental observation of the *m*/*z* 39 product, c−C_3_H_3_^+^, as the dominant pathway.

A confirmation of the proposed reaction pathways can be obtained from the data collected on the reactivity of C_2_D_5_^+^ with C_2_H_2_, by analyzing the extent of the H/D scrambling patterns in the mass spectra of [C_3_(H,D)_3_]^+^ and [C_4_(H,D)_5_]^+^ products. To avoid lengthening the main paper, this discussion is reported in Section 5 of the Supplementary Material.

### 4.3. Comparison with Previous Results

A direct comparison between our results and those in [39] should be made with care since reaction rates and BRs obtained using different mass spectrometric techniques may show some discrepancies, as previously outlined among Fourier Transform-ICR, selected ion flow drift tube methods and ion trap mass spectrometry [80,81,82]. One potential source of discrepancy is the different ranges of ion kinetic energies explored using different techniques. While in drift tube set-ups the ions have thermal translational and rotational energy distributions due to collisions with the buffer gas, a critical issue with early ICR experiments was the determination of the kinetic energy of the ions entering the ICR cell [83]. Due to the higher potentials at the trapping plates than at the center of the ICR cell, ions formed at or near the trapping plates were translationally excited with velocities characteristic of temperatures above thermal, but a quantitative assessment of the reactant ion kinetic energy was not always possible. In our guided ion beam technique, the kinetic energy of the ions entering the scattering cell is well defined and measurements are performed as a function of collision energy.

Another source of discrepancy may be related to an unknown amount of internal energy in the reactant ions as, in ICR experiments, ions can have different internal energies according to the ion generation and isolation method. In the previous study from literature [39], the C_2_H_5_^+^ ions were generated by electron ionization of C_2_H_5_Cl and C_2_H_6_ with a nominal electron energy of 16 eV, but inefficient internal relaxation prior to the reaction may have led to some portion of the reactant ions being generated in vibrationally excited states. In our experiment, the use of dissociative photoionization at well-defined photon energies allows for the control of internal excitation. As shown in Figure 2, BRs of the reaction products are strongly affected by the photon energy, it is therefore possible that the different BRs measured in that previous work [39] compared to this one are due to the presence of different amounts of internally excited C_2_H_5_^+^ ions. However, regardless of the source of the deviation between this work and previous literature, the calculated PES detailed in Section 3 and the MIKE spectra presented in Section 2.3, allow us to fully rationalize our experimental BRs.

## 5. Materials and Methods

### 5.1. Experimental Set-Up

The experimental set-up used for reactivity measurements has been described in detail elsewhere [55,56,84], and so, only a brief description is given here. The data on the reactivity of C_2_H_5_^+^ has been collected using the CERISES apparatus [85,86] in combination with the DESIRS beamline [87] of the SOLEIL synchrotron. CERISES is a guided ion beam tandem mass spectrometer consisting of two octopoles located between two quadrupole mass filters. This allows for the mass selection of reagent and product ions of ion-neutral reactions, with the neutral reagent being introduced in a scattering cell surrounding the final part of the first octopole.

The C_2_H_5_^+^/C_2_D_5_^+^ reagent ions used in this work were generated via VUV photoionization and fragmentation of C_2_H_5_Br (Sigma-Aldrich, 98% purity)/C_2_D_5_Br (Sigma-Aldrich, 99% purity with 99% deuteration), introduced into the source at approximately 1.8 × $10^{-6}$ mbar. The wide tuneability and intensity of the DESIRS VUV photon source allows for data to be collected as a function of the photon energy which, in turn, acts as a proxy for the internal energy of the reagent ions. The photon energies used were in the range of 10.4–14 eV, with a resolution of 20–40 meV defined by the monochromator slit setting. Photons of energies greater than 15.7 eV were removed by an Argon gas filter [88]. Photon energies in the absolute scale were obtained using the absorption lines of Argon around 11.823 and 14.304 eV [64,89], with systematic shifts of 10–20 meV above the tabulated values.

Neutral reagent (C_2_H_2_), Air Liquide, 99.7% purity) pressures used were of the order of 1 × $10^{-5}$ mbar, in order to ensure operation close to the single collision regime. In this way, we were able to reduce the contribution from secondary collisions and limit the attenuation of the parent beam to under 10%. As the C_2_H_2_ was dissolved in acetone (CH_3_C(O)CH_3_) as a stabilizer, the sample was first passed through a cryogenic trap containing dry ice in order to remove any acetone present before introduction into the experiment. Absolute pressures were measured using a MKS 398H differential manometer.

The collision energy available to the reactants depends on both the ionic charge (in this case +1) and the potential difference between the ion source and reaction cell. The retarding potential method [90] has been used to determine the maximum of the first derivative of the parent ion yield, the corresponding voltage of which defines the zero of the kinetic energy in the laboratory frame, which can then be converted into center-of-mass collision energies ($E_{CM}$). By changing the potentials of the reaction cell and all subsequent elements on the path of ions, we can scan a collision energy range from ≈ 0.07 to ≈ 6 eV in the center of mass frame. The FWHM of the collision energy in the scanned range is 0.16 eV in the $E_{CM}$ frame.

Data as a function of both photon and collision energies have been collected in the “multi-scan” mode where the signals for all ionic species of interest are collected at a given set of conditions before moving to the next one. In this way, we are able to drastically reduce any potential effects from drifts in source or reaction cell pressure. Absolute CS data rely on a calibration of the effective length of the reaction cell performed using the ArD^+^ product channel of the well-known reaction between Ar^+^ and D_2_ measured with a similar guided ion beam tandem mass spectrometer [74]. Cross sections are typically given with 10% errors resulting from the uncertainty in the absolute pressure measurement. However, for experimental conditions where ionic counting rates are small (close to appearance energy and/or channels with very small branching ratio), the uncertainty can exceed 10%. In such cases, a propagation of uncertainty is performed for all counting rates involved in the cross section measurement, applying a square root function on each counting measurement to derive its uncertainty.

Mass-analyzed ion kinetic energy (MIKE) spectra have been recorded using a ZAB2-SEQ tandem mass spectrometer of reverse double-focusing geometry, in which the magnetic sector precedes the electrostatic sector. Precursor ions (CH_2_C(CH_3_)CH_2_^+^/CD_2_C(CD_3_)CH_2_^+^) are prepared by dissociative electron ionization (70 eV, 150*C*) of CH_2_C(CH_3_)_2_ (Merck, 99.3% purity) and CD_2_C(CD_3_)_2_ (Merck, 99% purity and 99% deuteration), respectively. Following acceleration of 8 keV, precursor ions are mass-selected by the magnetic sector and allowed to dissociate spontaneously in the second field-free region of the instrument. The resultant ions are then analyzed by scanning the electrostatic sector. For peak profile measurements, the main beam width is reduced to 4 eV, corresponding to an energy resolution of 2000. For the *m*/*z* 55 ions (CH_2_C(CH_3_)CH_2_^+^), the metastable time window for ion fragmentation under these conditions is 9.4–18.3 μs, with the *m*/*z* 62 ions (CD_2_C(CD_3_)CD_2_^+^) having a metastable time window of 10.0–19.4 μs.

### 5.2. Computational Methodology

Electronic structure calculations to identify the underlying reaction mechanisms (i.e., stable intermediates and transition states) and aid in the interpretation of experimental results were performed with the Gaussian 16 suite of programs [71]. The ωB97X-D/cc-pVTZ level of theory, where ωB97X-D is a long-range corrected hybrid density functional that includes empirical atom–atom dispersion corrections [91] and cc-pVTZ refers to the correlation consistent polarized Valence Triple Zeta basis set (i.e., a triple-zeta basis set intended specifically for use in correlated calculations) was initially used for a preliminary mapping of the most relevant stationary points of the PES. Intrinsic reaction coordinate (IRC) calculations have then been performed to ensure that each TS connects to the relevant minima.

The accuracy of the relative energies obtained at the ωB97X-D/cc-pVTZ level of theory was tested against available literature values for the reaction enthalpies of the various product channels (as shown in Table 1 and in Table S1 of the Supplementary Material). Further accuracy tests were performed by calculating proton affinities of various organic molecules (ethyne, ethene, propene, butadiene, cyclobutene), as reported in Table S2 of the Supplementary Material. From such a comparison, it was concluded that the accuracy of the relative energies at the ωB97X-D/cc-pVTZ level of theory is not satisfactory, and thus, the relative energies were all recalculated using a composite method denoted as G4 [92] to obtain more accurate energies, and only the latter results are presented in Section 3.

Reaction enthalpies (Δ$H_{298}^{\circ }$) have also been calculated for comparison with literature values using both ωB97X-D/cc-pVTZ and G4 levels of theory.

## 6. Conclusions

Absolute cross sections and branching ratios for the reaction of the ethyl cation with ethyne have been measured as a function of collision energies, in the hyperthermal energy range (from ≈ 0.07 to ≈ 5 eV in the CM frame), under a single collision regime using a guided ion beam tandem mass spectrometer. The C_2_H_5_^+^ reactant ion is generated via dissociative ionization of C_2_H_5_Br using tunable VUV radiation in the energy range 10.4–14.0 eV. The two main product channels are [C_3_H_3_]^+^ plus CH_4_ (BR = 0.76±0.05) and [C_4_H_5_]^+^ plus H_2_ (BR = 0.22±0.02), where the reported values have been determined at a photon energy $E_{phot}$ = 11.3 eV and a collision energy in the CM frame $E_{CM}$ = 0.08 eV. At a higher internal excitation of C_2_H_5_^+^ and/or high collision energy, we observed an additional endothermic channel giving C_2_H_3_^+^ in association with either C_2_H_4_ or C_2_H_2_ plus H_2_.

Ab initio calculations of the most relevant stationary points on the potential energy surfaces allow for the rationalization of experimental results. Both channels stem from a common covalently bound intermediate, CH_3_CH_2_CHCH^+^, from which barrierless and exothermic pathways exist for the production of both cyclic and linear isomers of both main channels. From a comparison between the MIKE spectra of C(CH_3_)(CH_2_)_2_^+^ and C(CD_3_)(CD_2_)_2_^+^ and the calculated PES, we are able to conclude that, for both mass channels, the predominant isomer is the lower energy cyclic one, and that the contribution from the tunneling mechanisms is minimal.

However, the main result of this study is the re-evaluation of the product BRs for the two main reaction channels. Present experimental values, supported by theoretical calculations, indicate that that c−C_3_H_3_^+^ plus CH_4_ channel is the dominant one, at odds with previous literature data [39] where [C_4_H_5_]^+^ plus H_2_ was indicated as the primary product channel. Though the formation of c−C_3_H_2_(CH_3_)^+^ plus H_2_ is the most exothermic pathway, the barrier to fragmentation (−0.11 eV) is significantly higher than that required for c−C_3_H_3_^+^ formation (−1.06 eV), with the additional rearrangement required prior to fragmentation further enhancing the relative kinetic inhibition for this channel.

To assess the implications of our study on the modeling of Titan’s atmosphere, a sensitivity test was performed using the Titan photochemical model of V. Vuitton and collaborators [6] to estimate the changes in the density of ions and neutrals when the BRs for the title reaction are replaced with the present values. The photochemical model was run using the two different sets of BRs, as reported in Table 2, with the results summarized as follows. In each case, the densities that are reported are given as an integration over the atmospheric column, peaking around 1000 km and negligible below 700 km, as illustrated in Figure 39–42 of the cited work [6]:

- The density of [C_3_H_3_]^+^ ions is increased by ∼ 30% when our new BRs are used, despite the fact that the main formation reaction for [C_3_H_3_]^+^ in the model is C_2_H_4_^+^ + C_2_H_2_→ [C_3_H_3_]^+^ + CH_3_ and not the title reaction. This is a relevant difference that should be considered in light of the fact that a change in the abundance of [C_3_H_3_]^+^ can induce changes in other species, such as the recently detected c−C_3_H_2_ [43].
- The density of the [C_4_H_5_]^+^ ion decreases by about a factor of 2 when our new BRs are used. The repercussion on the production of [C_6_H_7_]^+^ is lower (with a maximum change of ∼ 20%), as the main formation reaction for this ion is [C_3_H_5_]^+^ + CH_3_CCH→ [C_6_H_7_]^+^ + H_2_ rather than [C_4_H_5_]^+^ plus C_2_H_4_. However, it still marks a significant change to the predicted density of this key astrochemical species in Titan’s environment.

Beyond the atmosphere of Titan, present results have implications for the chemical evolution of a range of different astrochemical environments due to the near-ubiquitous observation of both reactants. Notably, while the radioastronomical detection of the symmetric ion c−C_3_H_3_^+^ is difficult, its linear isomer H_2_CCCH^+^ has been very recently detected in a cold dense molecular cloud core [93], while both c−C_3_H_2_ and H_2_CCC isomers have long been detected in the ISM [94,95]. Finally, the observation of a third product, C_2_H_3_^+^, at high internal and collision energies, has implications for the modeling of high-energy plasmas.

## Figures and Tables

**Scheme 1:**
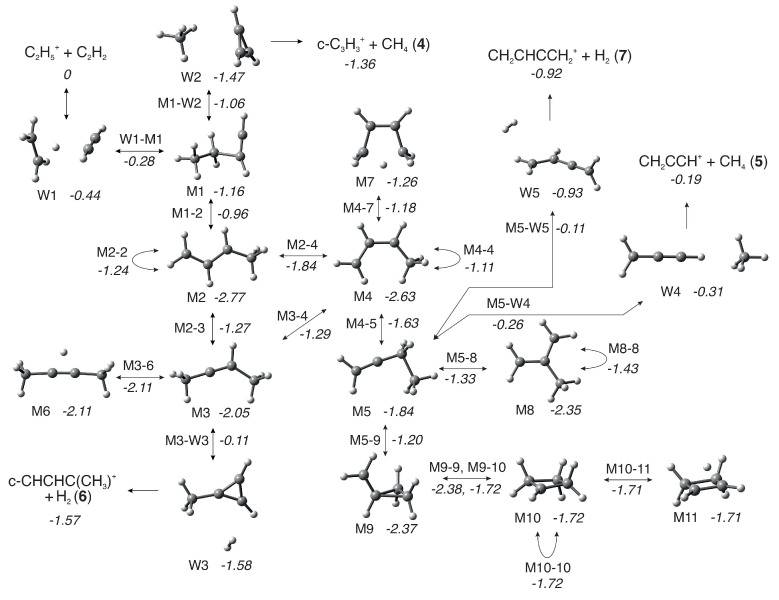
A sketch of the rearrangements and reaction pathways for the reaction of C_2_H_5_^+^ with C_2_H_2_ leading to product channels (4), (5), (6), and (7). The lettered labels of the different structures (e.g., **W1** ) and transition states (e.g., **M1-W2**) correspond to those described in the text and in Figure 8 and Figure 9.).

**Table 3:** Energy-dependent total rate constants ($k_{tot}$) measured at $E_{phot}=11.3$ eV and at various values of the average collision energy ($E_{ave}$).

*E_ave_*, eV	*k_tot_* ^1^	*k_tot_*/*k_L_* ^2^
0.096	1.7× $10^{-10}$	0.14
0.12	1.5× $10^{-10}$	0.13
0.52	4.3× $10^{-11}$	0.04
2.02	1.4× $10^{-10}$	0.12
4.52	5.2× $10^{-10}$	0.44

^1^ $k_{tot}$ is the total (i.e., summed over all the product channels) rate constant (in ${\mathrm{cm}}^{3}\cdot$${\mathrm{molecule}}^{-1}\cdot$$s^{-1}$) at the specified average collision energy ($E_{ave}$), estimated as detailed in the text. ^2^ $k_{L}$ is the Langevin rate constant for C_2_H_5_^+^ plus C_2_H_2_ equal to 1.18×$10^{-9}$ ${\mathrm{cm}}^{3}\cdot$${\mathrm{molecule}}^{-1}\cdot$$s^{-1}$.

## Data Availability

Data are contained within the article and Supplementary Materials.

## Supplementary Materials

The following supporting information can be downloaded at: https://www.mdpi.com/article/10.3390/molecules29040810/s1, Supplementary Material pdf file containing: Figures S1–S45 and Tables S1–S40; animated gif files: TS M1-W2.gif, TS M3-W3.gif, TS M5-W4.gif, TS M5-W5.gif 18 [58,69,70,96,97,98].
