# Supplementary material for: Reactivity of the Ethenium Cation (C2H5+) with Ethyne (C2H2): A Combined Experimental and Theoretical Study
Source: Molecules. 2024 Feb 9;29(4):810. doi: 10.3390/molecules29040810 (PMC10892252; doi:10.3390/molecules29040810)
Supplement: Supplementary file 1 [file molecules-29-00810-s001.zip › molecules-2813970-supplementary.pdf]

Supplementary Materials: Reactivity of the Ethenium Cation ($C_2H_5^+$) with Ethyne (C_2H_2): A Combined Experimental and Theoretical Study

Vincent Richardson [1,2], Miroslav Polášek [3], Claire Romanzin [4,5], Paolo Tosi [1], Roland Thissen [4,5], Christian Alcaraz [4,5], Ján Žabka [3] and Daniela Ascenzi [1,*]

1. Appearance Energy of $C_2H_4^{\bullet+}$ Fragment From C_2H_5Br

For the $C_2H_4^{+\bullet}$ fragment ion (*m/z* 28), we have measured an AE of 11.31 ± 0.33 eV, which is close to the calculated thermodynamic dissociation limit of 11.21 eV [1]. We note that, to the best of our knowledge, the AE of the $C_2H_4^{+\bullet}$ fragment has not previously been determined experimentally, with the observed AE close to the thermochemical threshold indicating an absence of energy barriers for the HBr elimination from $C_2H_5Br^+$. This is consistent with results from the equivalent process measured in the case of C_2H_5Cl, where the experimental AE for $C_2H_4^{+\bullet}$ (plus HCl) was observed well below the calculated barrier height for HCl loss [2]. Similarly, a PEPICO study of the dissociative photoionization of C_2H_5Cl [3] proposed a tunneling mechanism through the H-atom transfer barrier by determining dissociation rates as a function of internal energy of the parent ion for both energy-selected C_2H_5Cl and deuterated equivalents

2. Plots as a Function of the Collision Energy at Different Photon Energies

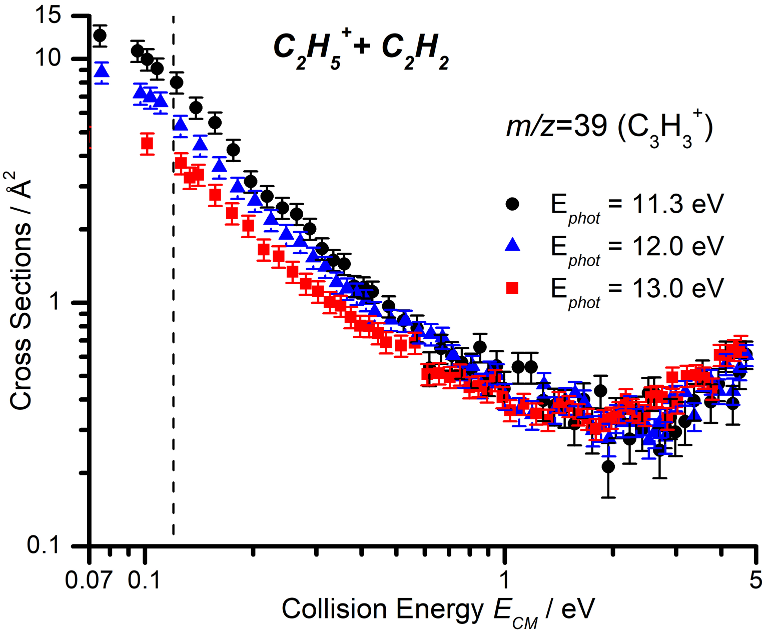

Figure S1. Cross sections as a function of E_{CM} for the *m/z* 39 product of the reaction of $C_2H_5^+$ with C_2H_2, at E_{phot} = 11.3, 12.0 and 13.0 eV. The dashed line represents the collision energy at which the corresponding scan as a function of the photon energy (Figure 2 of the main article) was conducted.

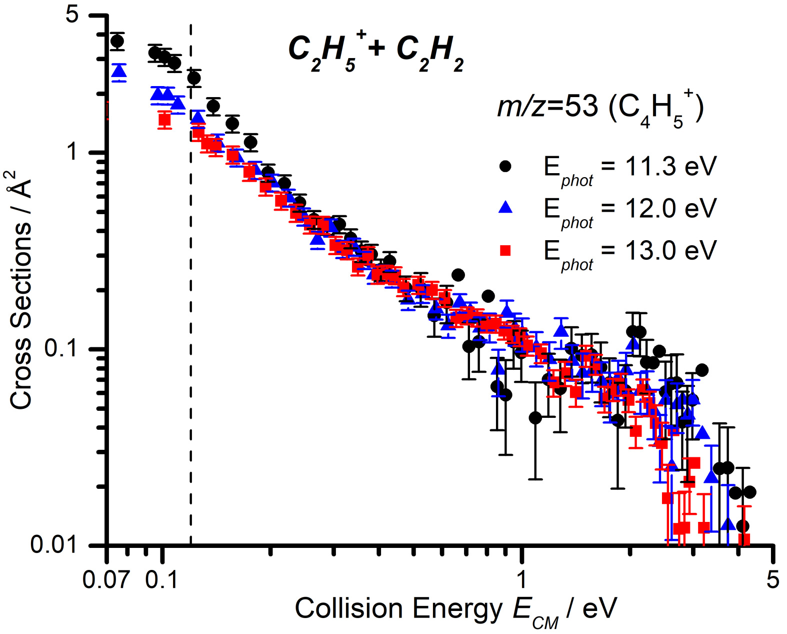

Figure S2. Cross sections as a function of E_{CM} for the *m/z* 53 product of the reaction of $C_2H_5^+$ with C_2H_2, at E_{phot} = 11.3, 12.0 and 13.0 eV. The dashed line represents the collision energy at which the corresponding scan as a function of the photon energy (Figure 2 of the main article) was conducted.

3. Test of the accuracy of the theoretical methods

The accuracy of energies calculated at the ωB97X-D/cc-pVTZ and G4 levels of theory was checked by:

i) calculation of reaction enthalpies, ΔH°_{298}, and comparison with data from [4] and/or [5], see Table S1;

ii) calculation of proton affinities for species similar to those of present study and comparison with data from [4] and/or [5], see Table S2;

Table S1. Comparison of calculated reaction enthalpies (ΔH°_{298}) at the ωB97X-D/cc-pVTZ and G4 level of theory with literature data estimated using thermochemical values taken from [4] and [5]. Values are reported in kJ/mol.

**Reaction channel**	**NIST** [4]	**ATcT** [5]	ωB97X-D/cc-pVTZ	**G4**
$C_2H_5^+$+C_2H_2 → c-$C_3H_3^+$+CH_4	-123.9	-130.4	-151.0	-131.4
$C_2H_5^+$+C_2H_2 → CH_2CCH^++CH_4	N/A	-15.0	-21.8	-16.2
$C_2H_5^+$+C_2H_2 → $C_2H_3^+$ + C_2H_4	+23.1	+36.8	+ 37.1	+35.8

Table S2. Comparison of calculated proton affinities at the ωB97X-D/cc-pVTZ and G4 level of theory with literature data from [4] and [5]. Values are reported in kJ/mol.

**Species**	**NIST** [4]	**ATcT** [5]	ωB97X-D/cc-pVTZ	**G4**
C_2H_2	641.4	642.7	664.2	645.1
C_2H_4	680.5	679.5	701.2	680.9
l-CH_2CHCH_3	751.6	744.2	777.4	744.4
l-$CH_2CHCHCH_2$	783.4	N/A	815.9	783.6
c-C_4H_6 cyclobutene	784.4	N/A	768.2	734.5

4. MIKES experiments

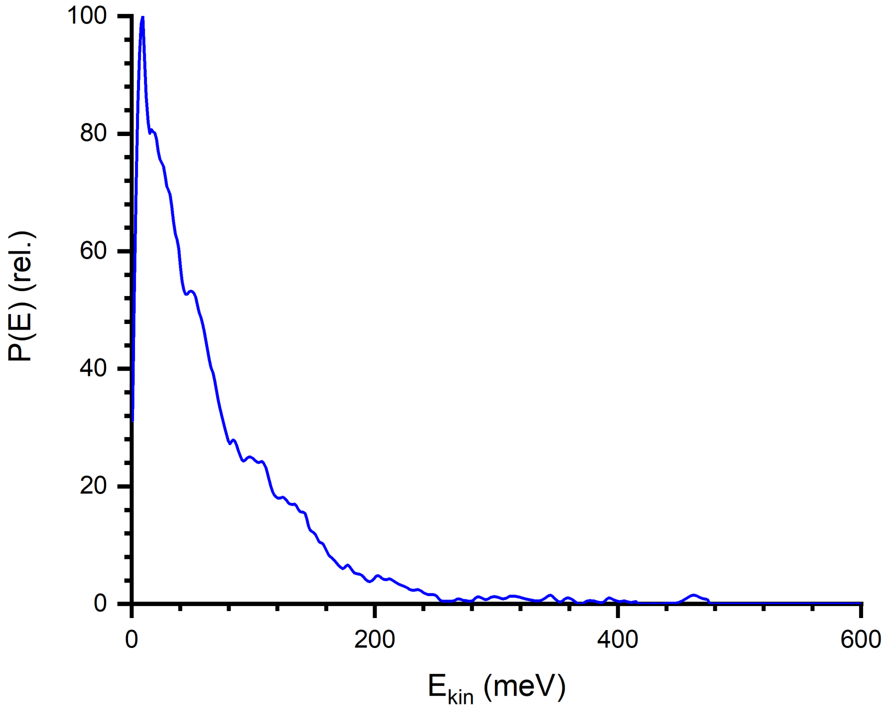

Figure S3. Kinetic energy release distribution of *m/z* 39 ion fragments ($[C_3H_3]^+$) from $CH_2C(CH_3)CH_2^+$

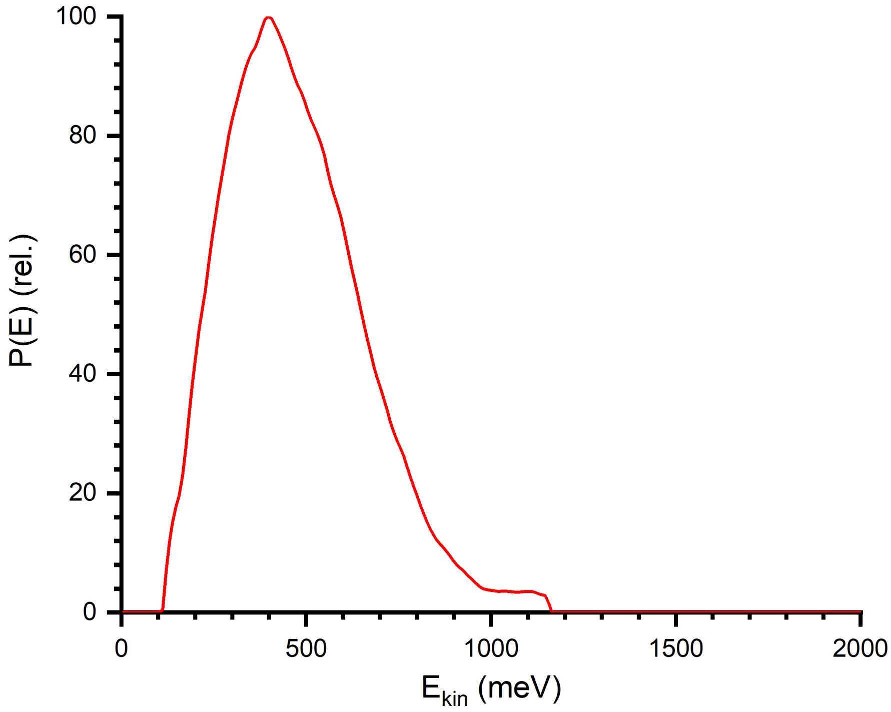

Figure S4. Kinetic energy release distribution of *m/z* 53 ion fragments ($[C_4H_5]^+$) from $CH_2C(CH_3)CH_2^+$

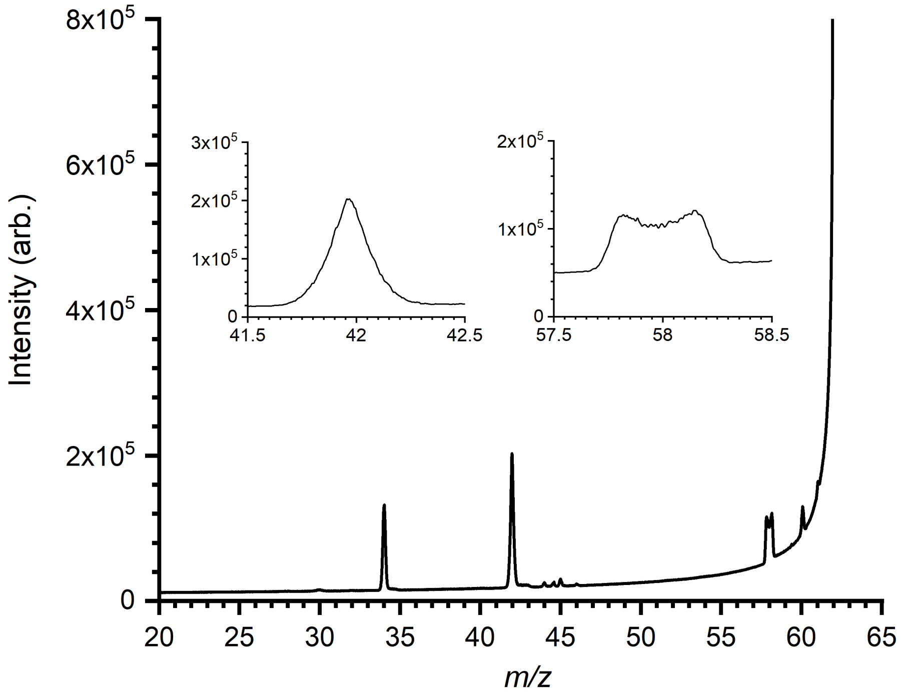

Figure S5. MIKE spectrum of the *m/z* 62 ion formed via dissociative ionization of d_8-2-methylpropane ($CD(CD_3)_3$). Insets show shapes of the *m/z* 42 (CD_4 loss) and 58 (D_2 loss) peaks, while the peak at *m/z* 34 corresponds to the ejection of C_2D_2.

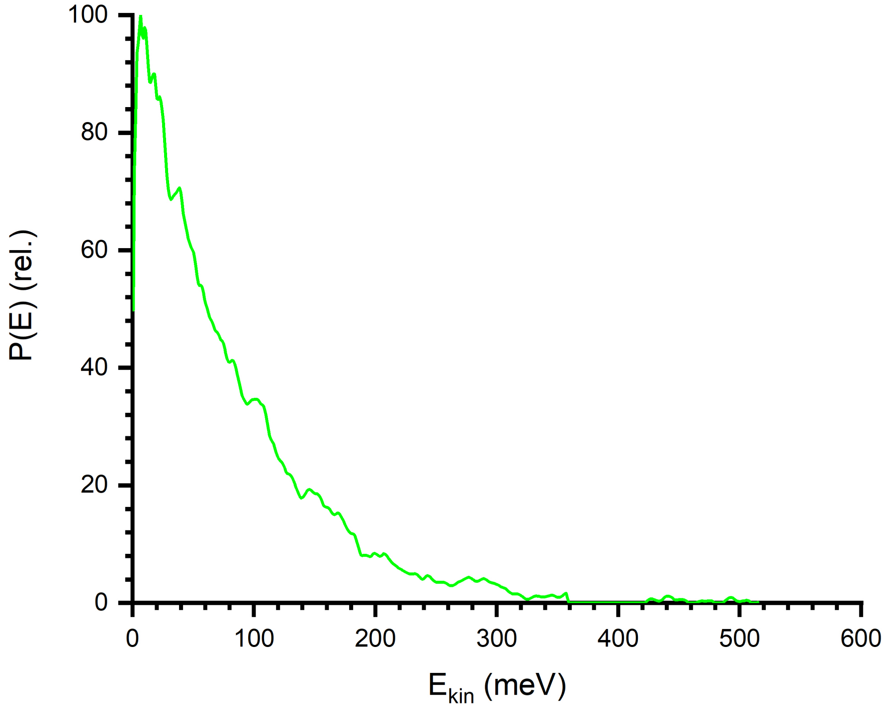

Figure S6. Kinetic energy release distribution of *m/z* 42 ion fragments ($[C_3D_3]^+$) from $CD_2C(CD_3)CD_2^+$

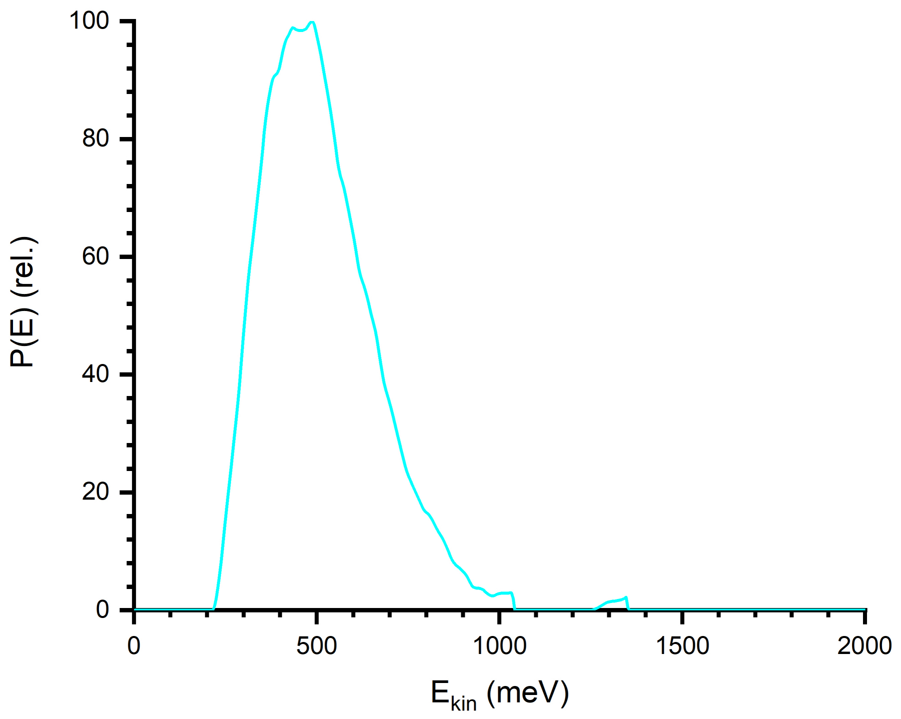

Figure S7. Kinetic energy release distribution of *m/z* 58 ion fragments ($[C_4D_5]^+$) from $CD_2C(CD_3)CD_2^+$

5. Results from IRC calculations on selected reaction pathways

IRC (intrinsic reaction coordinates) calculations performed at the G4 level of theory using the Gaussian 16 suite of programs are available as animated *gif* files and can be downloaded at https://www.mdpi.com/article/10.3390/molecules29040810/s1.

5.1. List of IRC calculations

- TS M1-W2.gif
- TS M3-W3.gif
- TS M5-W4.gif
- TS M5-W5.gif

6. Scrambling Discussion

Further insights on the reaction pathways can be obtained from the data collected on the reactivity of $C_2D_5^+$. In this case, depending on the extent of H/D scrambling, the $[C_3H_3]^+$ product appearing at *m/z* 39 for the reaction of $C_2H_5^+$ would give a mixture of $[C_3H_2D]^+$ (*m/z* 40), $[C_3HD_2]^+$ (*m/z* 41) and $[C_3D_3]^+$ (*m/z* 42), while the $[C_4H_5]^+$ product would shift from *m/z* 53 to a mixture of $[C_4H_2D_3]^+$ (*m/z* 56), $[C_4HD_4]^+$ (*m/z* 57) and $[C_4D_5]^+$ (*m/z* 58).

H/D scrambling in ion-molecule reactions involving organic ions is a well known process, with fragmentation from an intermediate complex which has undergone complete equilibration of H/D atoms over a given set of molecular sites producing a "statistical pattern" of relative abundances that can be calculated based on simple combinatorics [6]. Alternatively, if a "non-statistical pattern" is observed, that that can allow us to infer mechanistic information on the underlying dynamics. In the case of covalently bound complexes (as for the intermediates **M1**, **M2**, **M3** etc...) complete energy randomization is expected once the complexes are formed and, since hydrogen atoms can migrate along the carbon backbone, the distribution of H/D isotopic scrambled products is expected to be statistical. Comparison of the experimental BRs with those calculated assuming complete scrambling of all the H/D atoms is given in Table S3, while Fig. S8 shows the mass spectra from which experimental BRs have been obtained.

Table S3. Experimental and statistical BRs for the *m/z* 40, 41, 42, 56, 57 and 58 products of the reaction of $C_2D_5^+$ with C_2H_2 at E_{phot} = 11.3 eV and E_{CM} = 0.08 eV.

Product Mass (*m/z*)	Ion+neutral products	BR, Exp.	BR, Stat.
40	$C_3H_2D^+$+CD_4	0.17±0.02	0.143
41	$C_3HD_2^+$+CD_3H	0.56±0.03	0.571
42	$C_3D_3^+$+CD_2H_2	0.28±0.02	0.286
56	$C_4H_2D_3^+$+D_2	0.41±0.02	0.476
57	$C_4HD_4^+$+HD	0.53±0.03	0.476
58	$C_4D_5^+$+H_2	0.06±0.01	0.048

In the case of $[C_3(H,D)_3]^+$ the observed BRs are fully compatible with the statistical pattern, while for the $[C_4(H,D)_5]^+$ product, there seems to be a slight preference for HD loss (*m/z* 57) rather than a complete H/D scrambling. However, given that these BRs have been obtained from individual mass spectra, we believe that the observed results are consistent with a statistical pattern within the experimental precision of the measurement.

Rationalisation of the observed scrambling is possible when one considers the possible mechanisms of H/D scrambling illustrated in the computational scheme included in the main text, which includes intermediates and TSs relevant to the rearrangements of H/D atoms via the interconversion of the $[C_4H_7^+]$ adducts. The relative energies of all additional minima and transition states are either lower than or comparable with the energies of the transition states leading to dissociation into c-$C_3H_3^+$ plus CH_4 (**M1-W2**) and c-$C_3H_2(CH_3)^+$ plus H_2 (**M3-W3**).

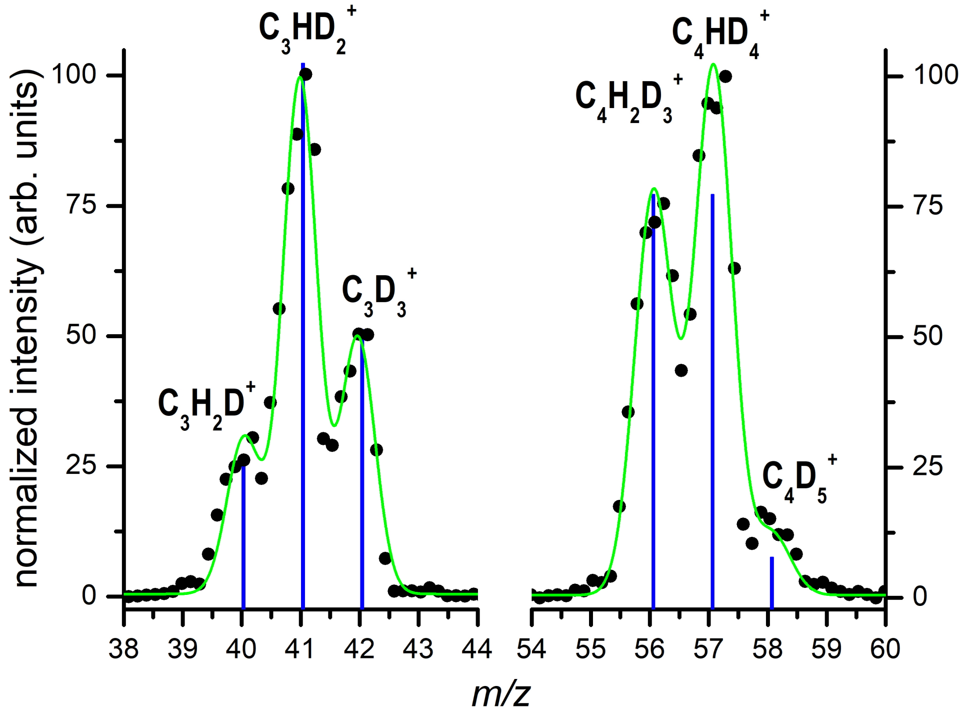

Figure S8. Mass spectra from the reaction of $C_2D_5^+$ with C_2H_2 in the regions of formation of $C_3(H,D)_3^+$ and $C_4(H,D)_5^+$ ion products taken at E_{phot} = 11.3 eV and E_{CM} = 0.08 eV. *Black dots* are the experimental values (averages of three repeats), *green lines* are the peaks' best fits assuming Gaussian functions with a width of 0.6 and *blue bars* are the calculated statistical patterns.

7. Structures and geometries (atomic coordinates) of the intermediates and transition states

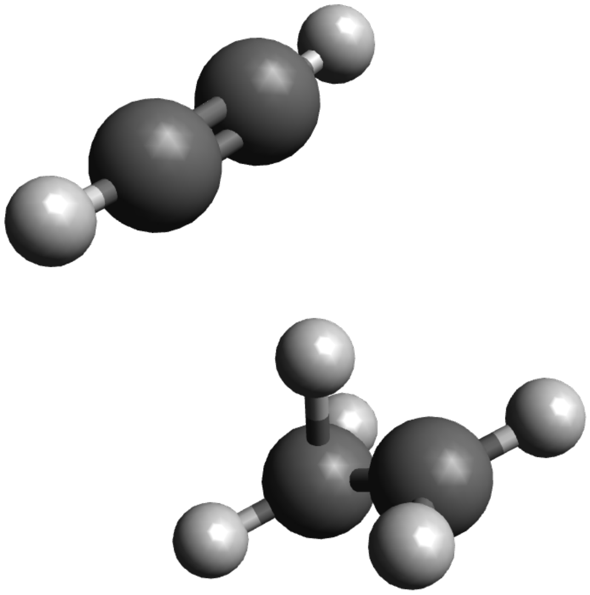

Figure S9. Structure diagram for the ion/induced dipole complex **W1** optimized at the G4 level of theory

Table S4. Atom coordinates for the ion/induced dipole complex **W1** in angstroms (Å) optimized at the G4 level of theory

Atom Type	X Coordinate (Å)	Y Coordinate (Å)	Z Coordinate (Å)
C_1	0.680503	0.000000	-1.500532
C_2	-0.680503	0.000000	-1.500532
C_3	0.000000	-0.599149	1.730883
C_4	0.000000	0.599149	1.730883
H_1	1.230845	-0.939427	-1.509170
H_2	1.230845	0.939427	-1.509170
H_3	-1.230845	-0.939427	-1.509170
H_4	-1.230845	0.939427	-1.509170
H_5	0.000000	0.000000	-0.309951
H_6	0.000000	1.664129	1.791213
H_7	0.000000	-1.664129	1.791213

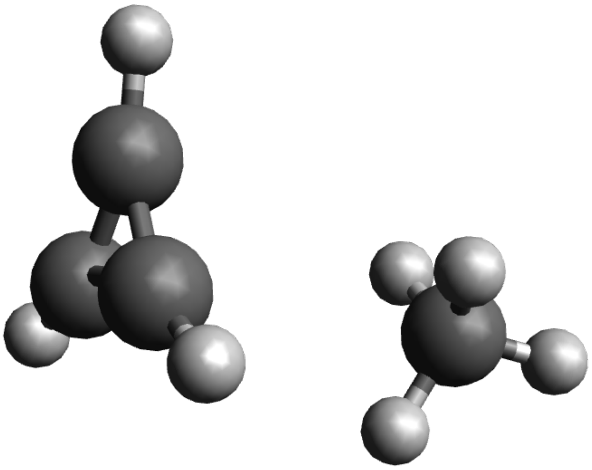

Figure S10. Structure diagram for the ion/induced dipole complex **W2** optimized at the G4 level of theory

Table S5. Atom coordinates for the ion/induced dipole complex **W2** in angstroms (Å) optimized at the G4 level of theory

Atom Type	X Coordinate (Å)	Y Coordinate (Å)	Z Coordinate (Å)
C_1	-2.310674	-0.000629	-0.492837
C_2	0.549742	-0.251440	0.825837
C_3	1.007508	0.766224	0.057443
C_4	1.294904	-0.513831	-0.274730
H_1	-2.185190	0.589026	0.415337
H_2	-3.369894	-0.035553	-0.732048
H_3	-1.780776	0.464141	-1.323512
H_4	-1.960032	-1.022546	-0.345553
H_5	-0.001079	-0.596986	1.686276
H_6	1.081554	1.823740	-0.143178
H_7	1.765638	-1.224614	-0.936115

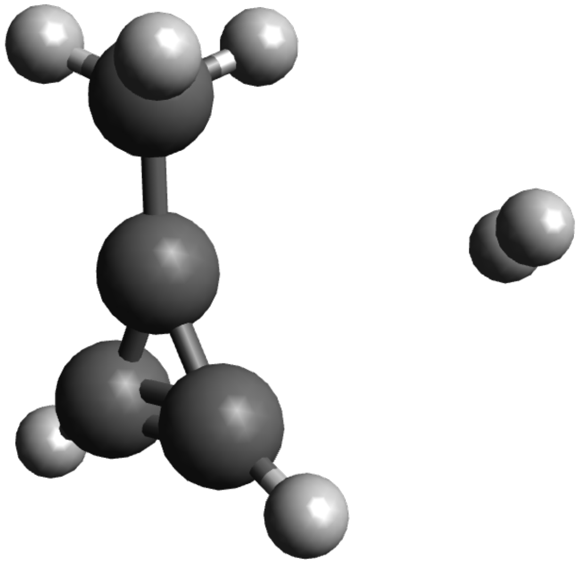

Figure S11. Structure diagram for the ion/induced dipole complex **W3** optimized at the G4 level of theory

Table S6. Atom coordinates for the ion/induced dipole complex **W3** in angstroms (Å) optimized at the G4 level of theory

Atom Type	X Coordinate (Å)	Y Coordinate (Å)	Z Coordinate (Å)
C_1	-1.680051	-0.134694	0.056181
C_2	-0.240611	0.061366	-0.065454
C_3	0.869786	0.844133	0.059079
C_4	0.998066	-0.426048	-0.371907
H_1	-2.053475	-0.698345	-0.798079
H_2	-2.205524	0.808719	0.168405
H_3	-1.852241	-0.749733	0.945127
H_4	1.596921	-2.238186	1.927375
H_5	1.326644	1.788022	0.311442
H_6	2.186591	-1.788138	1.998849
H_7	1.630582	-1.228649	-0.715695

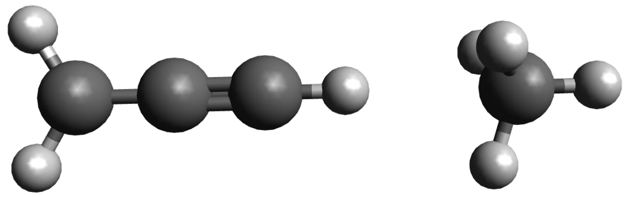

Figure S12. Structure diagram for the weakly bound adduct **W4** optimized at the G4 level of theory

Table S7. Atom coordinates for the weakly bound adduct **W4** in angstroms (Å) optimized at the G4 level of theory.

Atom Type	X Coordinate (Å)	Y Coordinate (Å)	Z Coordinate (Å)
C_1	0.000000	0.198077	0.000000
C_2	-0.032564	1.426396	0.000000
C_3	-0.068317	2.767990	0.000000
C_4	0.073734	-3.205044	0.000000
H_1	0.853333	3.352476	0.000000
H_2	-1.019784	3.302566	0.000000
H_3	0.029056	-0.882346	0.000000
H_4	0.584521	-2.851452	0.901274
H_5	0.108005	-4.295307	0.000000
H_6	-0.976769	-2.898993	0.000000
H_7	0.584521	-2.851452	-0.901274

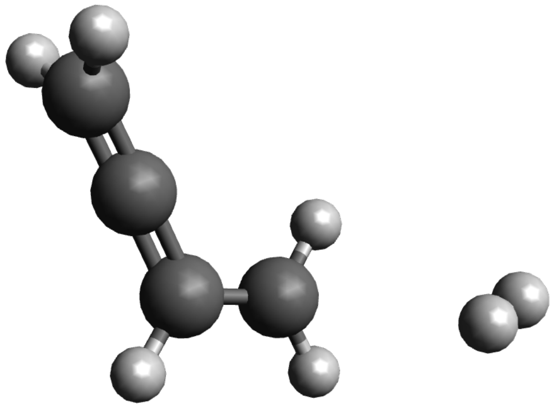

Figure S13. Structure diagram for the weakly bound adduct **W5** optimized at the G4 level of theory

Table S8. Atom coordinates for the weakly bound adduct **W5** in angstroms (Å) optimized at the G4 level of theory.

Atom Type	X Coordinate (Å)	Y Coordinate (Å)	Z Coordinate (Å)
C_1	-1.044183	-0.531695	0.479436
C_2	0.001464	-0.919949	-0.316558
C_3	1.239038	-0.380477	-0.089014
C_4	2.400676	0.110357	0.110067
H_1	-2.827936	1.830440	-0.214597
H_2	-2.525516	1.635126	-0.866390
H_3	-2.039337	-0.945633	0.339173
H_4	-0.906933	0.192131	1.2774441
H_5	-0.127783	-1.645970	-1.115305
H_6	2.726613	1.017318	-0.404059
H_7	3.103893	-0.361649	0.799801

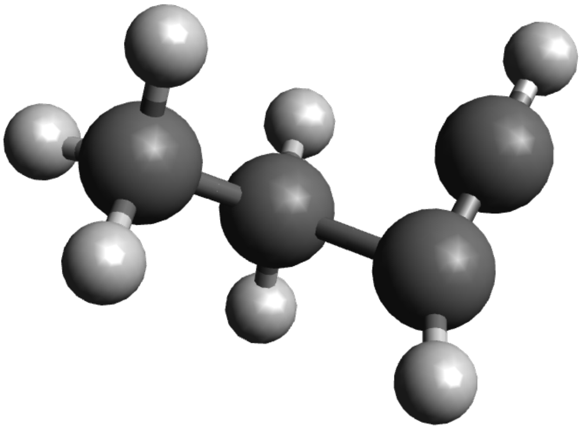

Figure S14. Structure diagram for the intermediate structure **M1** optimized at the G4 level of theory

Table S9. Atom coordinates for the intermediate structure **M1** in angstroms (Å) optimized at the G4 level of theory

Atom Type	X Coordinate (Å)	Y Coordinate (Å)	Z Coordinate (Å)
C_1	-1.602067	-0.009819	-0.271740
C_2	-0.361927	-0.179622	0.579648
C_3	0.869302	0.670483	-0.102644
C_4	1.397379	-0.431943	-0.234191
H_1	-2.429783	-0.505531	0.237078
H_2	-1.478231	-0.469785	-1.251100
H_3	-1.852922	1.041777	-0.400444
H_4	-0.151116	-1.244593	0.757634
H_5	-0.368092	0.264212	1.574885
H_6	0.828413	1.737407	-0.217618
H_7	1.837329	-1.403990	-0.350621

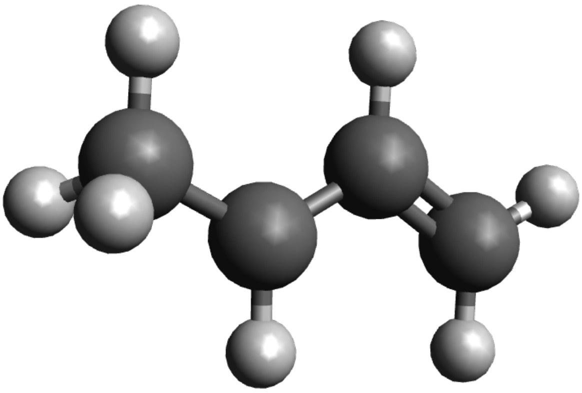

Figure S15. Structure diagram for the intermediate structure **M2** optimized at the G4 level of theory

Table S10. Atom coordinates for the intermediate structure **M2** in angstroms (Å) optimized at the G4 level of theory

Atom Type	X Coordinate (Å)	Y Coordinate (Å)	Z Coordinate (Å)
C_1	0.310374	-0.042517	0.000000
C_2	0.823716	0.683439	1.257405
C_3	2.363714	0.680983	1.258814
C_4	3.080343	1.238165	2.225273
H_1	0.667029	-1.051327	0.000000
H_2	0.667047	0.461881	-0.873652
H_3	-0.759626	-0.042504	0.000000
H_4	0.205958	1.121739	2.013162
H_5	2.820301	0.180366	0.394468
H_6	4.178608	1.236429	2.226268
H_7	2.623756	1.738777	3.089620

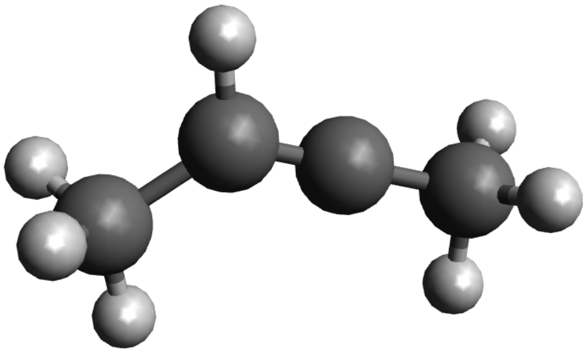

Figure S16. Structure diagram for the intermediate structure **M3** optimized at the G4 level of theory

Table S11. Atom coordinates for the intermediate structure **M3** in angstroms (Å) optimized at the G4 level of theory

Atom Type	X Coordinate (Å)	Y Coordinate (Å)	Z Coordinate (Å)
C_1	0.310374	-0.042517	0.000000
C_2	0.823716	0.683439	1.257405
C_3	2.363714	0.680983	1.258814
C_4	2.874729	-0.771759	1.260762
H_1	0.667029	-1.051327	0.000000
H_2	0.667047	0.461881	-0.873652
H_3	-0.759626	-0.042504	0.000000
H_4	0.205958	1.121739	2.013162
H_5	2.919192	-1.135556	0.255489
H_6	3.851688	-0.805611	1.695855
H_7	2.208364	-1.383483	1.832296

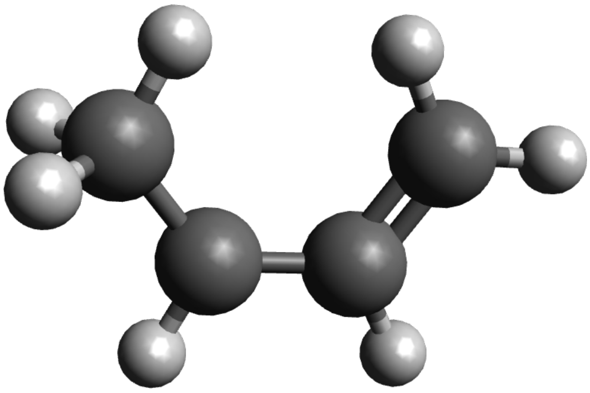

Figure S17. Structure diagram for the intermediate structure **M4** optimized at the G4 level of theory

Table S12. Atom coordinates for the intermediate structure **M4** in angstroms (Å) optimized at the G4 level of theory

Atom Type	X Coordinate (Å)	Y Coordinate (Å)	Z Coordinate (Å)
C_1	0.310374	-0.042517	0.000000
C_2	0.823716	0.683439	1.257405
C_3	2.363714	0.680983	1.258814
C_4	3.080313	0.114079	0.298003
H_1	0.667029	-1.051327	0.000000
H_2	0.667047	0.461881	-0.873652
H_3	-0.759626	-0.042504	0.000000
H_4	0.205958	1.121739	2.013162
H_5	2.820328	1.186801	2.120111
H_6	4.178578	0.112311	0.299017
H_7	2.623699	-0.391736	-0.563297

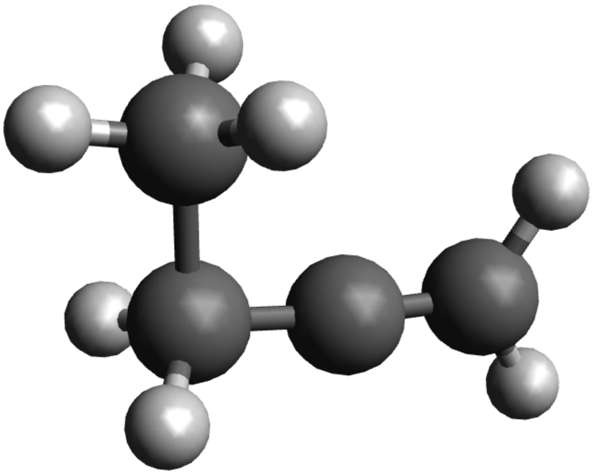

Figure S18. Structure diagram for the intermediate structure **M5** optimized at the G4 level of theory

Table S13. Atom coordinates for the intermediate structure **M5** in angstroms (Å) optimized at the G4 level of theory

Atom Type	X Coordinate (Å)	Y Coordinate (Å)	Z Coordinate (Å)
C_1	0.310374	-0.042517	0.000000
C_2	0.823716	0.683439	1.257405
C_3	2.363714	0.680983	1.258814
C_4	3.080343	1.238165	2.225273
H_1	0.667029	-1.051327	0.000000
H_2	0.667047	0.461881	-0.873652
H_3	-0.759626	-0.042504	0.000000
H_4	0.468660	1.692812	1.256428
H_5	2.820301	0.180366	0.394468
H_6	4.178608	1.236429	2.226268
H_7	0.465447	0.180171	2.131055

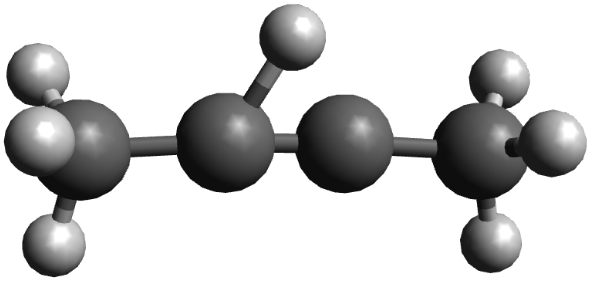

Figure S19. Structure diagram for the intermediate structure **M6** optimized at the G4 level of theory

Table S14. Atom coordinates for the intermediate structure **M6** in angstroms (Å) optimized at the G4 level of theory

Atom Type	X Coordinate (Å)	Y Coordinate (Å)	Z Coordinate (Å)
C_1	2.067305	0.062682	-0.000064
C_2	0.613307	-0.022204	-0.000044
C_3	-0.614001	-0.017096	0.000048
C_4	-2.067510	0.061156	0.000010
H_1	2.480072	-0.400725	0.894013
H_2	2.321056	1.123685	-0.000180
H_3	2.480119	-0.400976	-0.893990
H_4	0.005776	-1.147039	0.000277
H_5	-2.323483	1.122057	-0.000095
H_6	-2.479057	-0.402326	-0.894559
H_7	-2.479086	-0.401899	0.894836

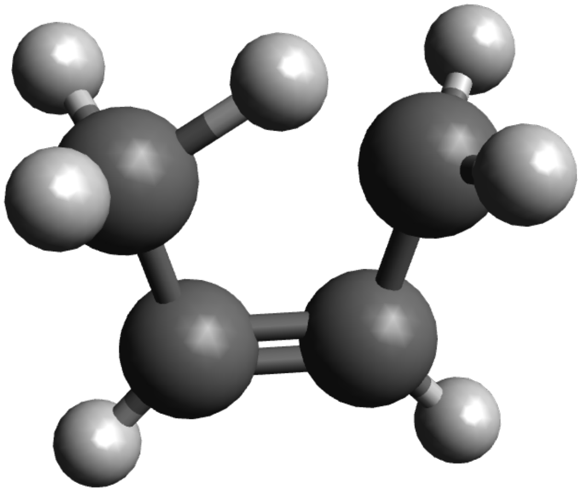

Figure S20. Structure diagram for the intermediate structure **M7** optimized at the G4 level of theory

Table S15. Atom coordinates for the intermediate structure **M7** in angstroms (Å) optimized at the G4 level of theory

Atom Type	X Coordinate (Å)	Y Coordinate (Å)	Z Coordinate (Å)
C_1	0.000000	-1.066746	-0.622575
C_2	0.000000	-0.657601	0.810293
C_3	0.000000	0.657601	0.810293
C_4	0.000000	1.066746	-0.622575
H_1	0.000000	0.000000	-1.321913
H_2	-0.913578	-1.487904	-1.044991
H_3	0.913578	-1.487904	-1.044991
H_4	0.000000	-1.363352	1.626129
H_5	0.000000	1.363352	1.626129
H_6	-0.913578	1.487904	-1.044991
H_7	0.913578	1.487904	-1.044991

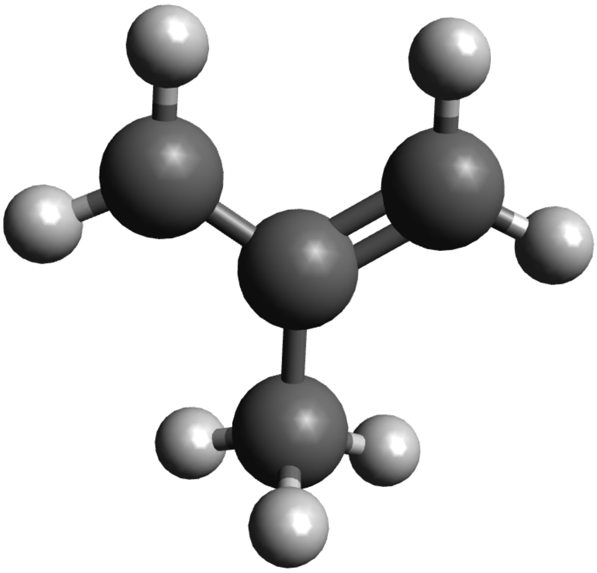

Figure S21. Structure diagram for the intermediate structure **M8** optimized at the G4 level of theory

Table S16. Atom coordinates for the intermediate structure **M8** in angstroms (Å) optimized at the G4 level of theory

Atom Type	X Coordinate (Å)	Y Coordinate (Å)	Z Coordinate (Å)
C_1	1.780848	-0.085034	0.000000
C_2	0.425648	-0.085034	0.000000
C_3	-0.290976	1.030539	-0.000044
C_4	-0.214595	-1.485637	0.000087
H_1	2.374433	-1.009072	-0.000019
H_2	2.374464	0.838980	0.000026
H_3	-1.389242	1.030539	-0.000025
H_4	0.165619	2.029390	-0.000110
H_5	0.328366	-2.126040	-0.663221
H_6	-0.186023	-1.890801	0.989999
H_7	-1.230973	-1.413215	-0.326458

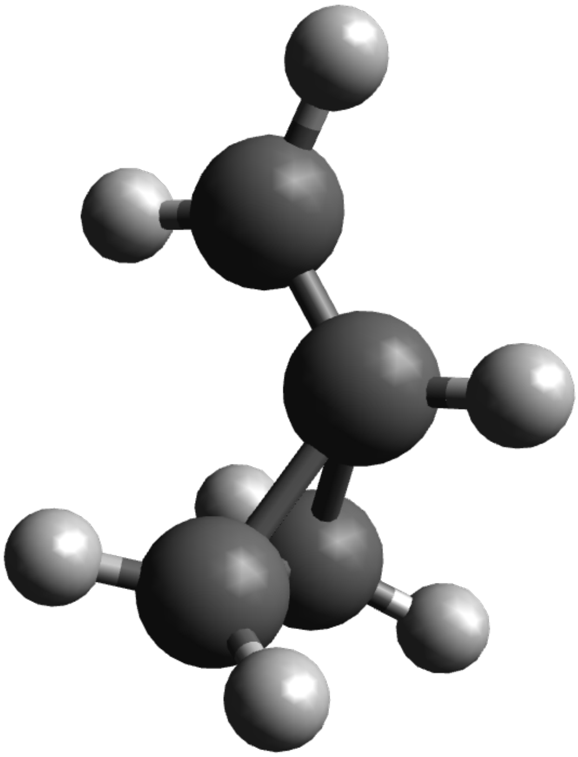

Figure S22. Structure diagram for the intermediate structure **M9** optimized at the G4 level of theory

Table S17. Atom coordinates for the intermediate structure **M9** in angstroms (Å) optimized at the G4 level of theory

Atom Type	X Coordinate (Å)	Y Coordinate (Å)	Z Coordinate (Å)
C_1	0.947248	-0.704994	0.142310
C_2	0.947633	0.704394	0.143651
C_3	-0.383762	0.000945	-0.530778
C_4	-1.507495	-0.000185	0.210231
H_1	1.444236	-1.251978	-0.647581
H_2	-0.409160	0.002124	-1.633746
H_3	0.728960	-1.254982	1.045756
H_4	0.729719	1.252606	1.048268
H_5	-2.487174	0.000076	-0.254342
H_6	-1.473483	-0.001408	1.294232
H_7	1.445157	1.252600	-0.645073

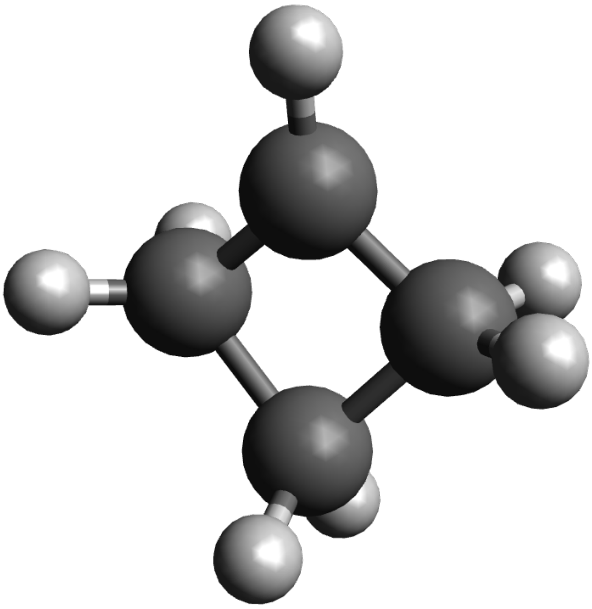

Figure S23. Structure diagram for the intermediate structure **M10** optimized at the G4 level of theory

Table S18. Atom coordinates for the intermediate structure **M10** in angstroms (Å) optimized at the G4 level of theory

Atom Type	X Coordinate (Å)	Y Coordinate (Å)	Z Coordinate (Å)
C_1	0.000000	0.000000	-1.066368
C_2	-0.251010	1.050006	0.077813
C_3	0.000000	0.000000	1.041390
C_4	-0.276153	-1.078702	0.013687
H_1	-0.796874	0.131131	-1.790601
H_2	0.949645	-0.105139	-1.580018
H_3	-1.169197	1.615889	0.296592
H_4	0.535382	1.819897	0.074858
H_5	-1.227551	-1.631327	-0.010864
H_6	0.475713	-1.861746	0.194503
H_7	0.139410	0.008278	2.120716

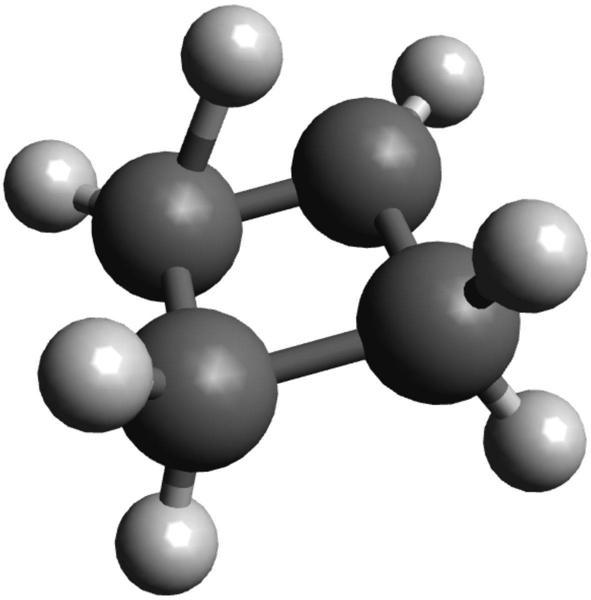

Figure S24. Structure diagram for the intermediate structure **M11** optimized at the G4 level of theory

Table S19. Atom coordinates for the intermediate structure **M11** in angstroms (Å) optimized at the G4 level of theory

Atom Type	X Coordinate (Å)	Y Coordinate (Å)	Z Coordinate (Å)
C_1	0.067017	-0.805632	0.777646
C_2	0.007091	0.736408	0.766111
C_3	0.007091	0.736408	-0.766111
C_4	0.067017	-0.805632	-0.777646
H_1	0.895450	1.227553	1.215234
H_2	-0.916327	1.157123	1.216061
H_3	0.895450	1.227553	-1.215234
H_4	-0.916327	1.157123	-1.216061
H_5	0.096729	-1.578908	-1.516394
H_6	0.096729	-1.578908	1.516394
H_7	-1.041001	-0.780851	0.000000

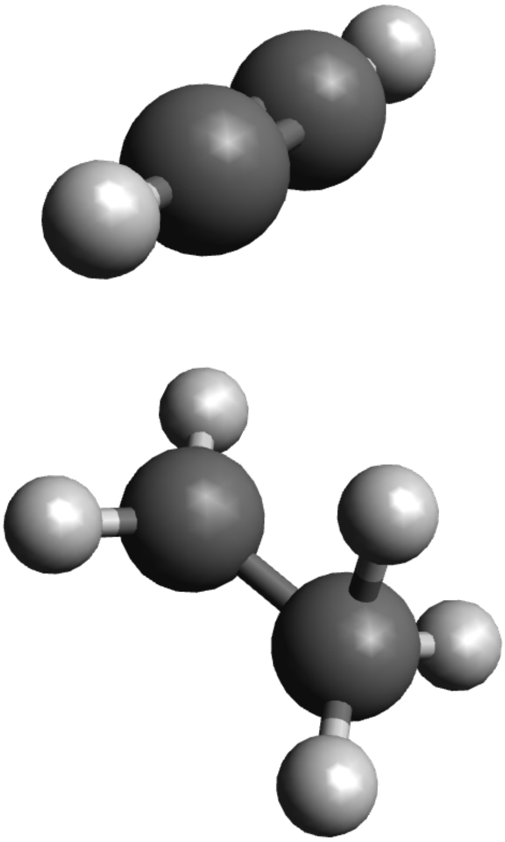

Figure S25. Structure diagram for the transition state **W1-M1** optimized at the G4 level of theory

Table S20. Atom coordinates for the transition state **W1-M1** in angstroms (Å) optimized at the G4 level of theory

Atom Type	X Coordinate (Å)	Y Coordinate (Å)	Z Coordinate (Å)
C_1	-1.135485	-1.147266	-0.383385
C_2	0.136499	-1.113177	0.170930
C_3	0.697141	1.787907	0.783771
C_4	1.183280	1.676273	-0.304731
H_1	-1.994868	-1.250381	0.269246
H_2	-1.251750	-1.419905	-1.426118
H_3	-0.877595	0.004000	-0.385828
H_4	1.025403	-1.146610	-0.449449
H_5	0.286213	-0.980121	1.236759
H_6	0.290166	1.949884	1.756255
H_7	1.640997	1.639396	-1.267450

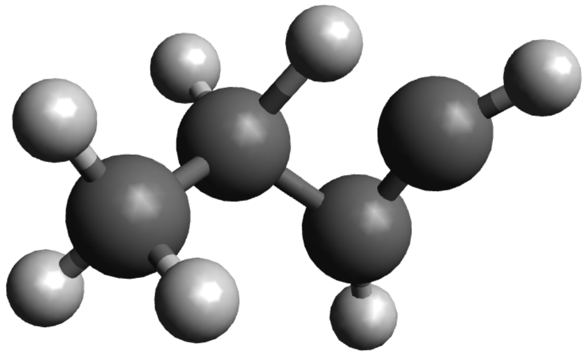

Figure S26. Structure diagram for the transition state **M1-2** optimized at the G4 level of theory

Table S21. Atom coordinates for the transition state **M1-2** in angstroms (Å) optimized at the G4 level of theory

Atom Type	X Coordinate (Å)	Y Coordinate (Å)	Z Coordinate (Å)
C_1	-0.461148	-0.895152	-0.950771
C_2	-0.223777	-0.265423	0.395829
C_3	-0.253312	1.226573	0.553452
C_4	0.998911	0.988929	0.677169
H_1	-0.065167	-1.909579	-0.993785
H_2	-0.040972	-0.293274	-1.756786
H_3	-1.546145	-0.946578	-1.086041
H_4	1.042381	-0.333266	0.624461
H_5	-0.511450	-0.819599	1.290604
H_6	-0.990593	2.010814	0.501023
H_7	2.051272	1.236557	0.744846

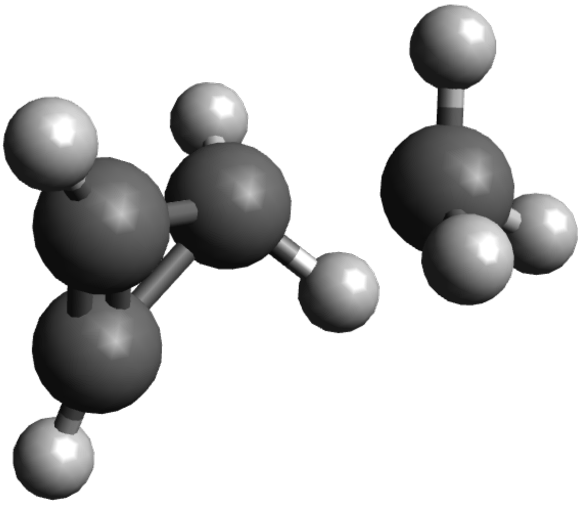

Figure S27. Structure diagram for the transition state **M1-W2** optimized at the G4 level of theory

Table S22. Atom coordinates for the transition state **M1-W2** in angstroms (Å) optimized at the G4 level of theory

Atom Type	X Coordinate (Å)	Y Coordinate (Å)	Z Coordinate (Å)
C_1	-1.286214	-0.345014	-0.301522
C_2	0.267745	0.477457	-0.254724
C_3	1.357125	-0.515634	0.055081
C_4	1.295765	0.430782	0.897539
H_1	-1.286544	-0.579549	-1.361420
H_2	-2.179021	0.243180	-0.063214
H_3	-1.194215	-1.218056	0.338091
H_4	-0.665675	0.669974	0.407787
H_5	0.318751	1.199203	-1.061819
H_6	1.787669	-1.392681	-0.400421
H_7	1.584614	1.030337	1.744621

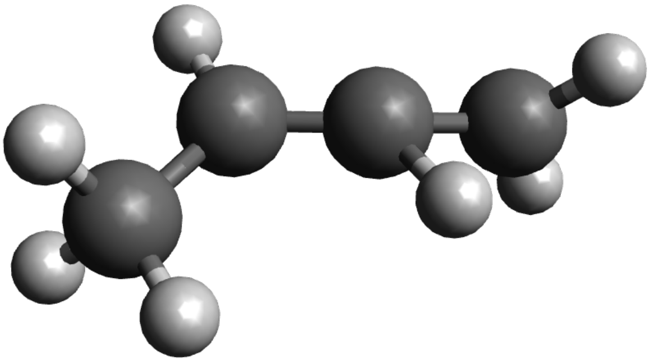

Figure S28. Structure diagram for the transition state **M2-3** optimized at the G4 level of theory

Table S23. Atom coordinates for the transition state **M2-3** in angstroms (Å) optimized at the G4 level of theory

Atom Type	X Coordinate (Å)	Y Coordinate (Å)	Z Coordinate (Å)
C_1	1.000388	-1.134110	-0.874426
C_2	-0.588347	0.238432	-0.472364
C_3	0.314953	0.546437	0.438915
C_4	1.347453	1.190160	1.047578
H_1	-0.384255	-1.910459	-0.422170
H_2	-0.947369	-1.212021	-1.963447
H_3	-2.046127	-1.294109	-0.597065
H_4	-1.127111	1.084073	-0.899737
H_5	0.829079	-0.289568	1.114929
H_6	2.249882	1.464899	0.497390
H_7	1.352228	1.316267	2.130396

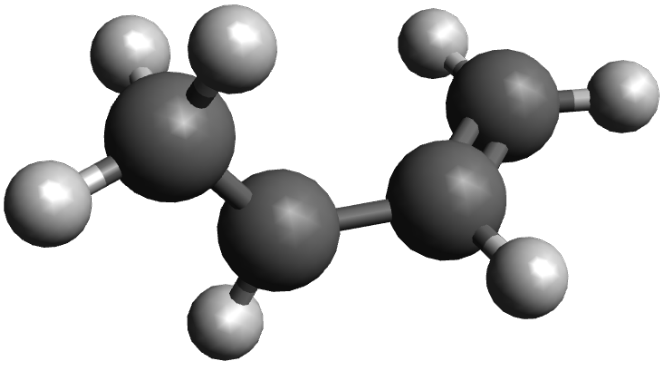

Figure S29. Structure diagram for the transition state **M2-4** optimized at the G4 level of theory

Table S24. Atom coordinates for the transition state **M2-4** in angstroms (Å) optimized at the G4 level of theory

Atom Type	X Coordinate (Å)	Y Coordinate (Å)	Z Coordinate (Å)
C_1	1.750046	-0.387221	0.040992
C_2	0.594568	0.521146	-0.135432
C_3	-0.709417	0.170909	0.018611
C_4	-1.938804	-0.237863	-0.031332
H_1	2.196902	-0.470533	-0.956884
H_2	1.463707	-1.375307	0.386796
H_3	2.507212	0.059872	0.683196
H_4	-0.217380	0.636613	1.043645
H_5	-2.328413	-0.608244	-0.972222
H_6	-2.589855	-0.211786	0.831001
H_7	0.789471	1.567555	-0.372568

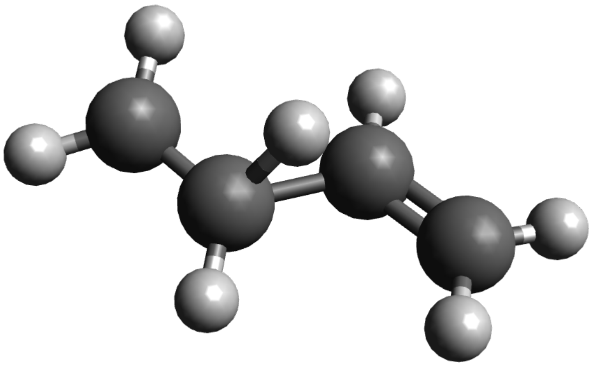

Figure S30. Structure diagram for the transition state **M2-2** optimized at the G4 level of theory

Table S25. Atom coordinates for the transition state **M2-2** in angstroms (Å) optimized at the G4 level of theory

Atom Type	X Coordinate (Å)	Y Coordinate (Å)	Z Coordinate (Å)
C_1	1.863548	0.132541	0.011462
C_2	0.663190	-0.444748	-0.002780
C_3	-0.535311	0.372419	0.029270
C_4	-1.831114	-0.109379	0.075952
H_1	1.983049	1.209143	0.013485
H_2	-1.165831	0.215954	-0.965810
H_3	2.766552	-0.461640	0.033442
H_4	0.549740	-1.521336	-0.004204
H_5	-0.405618	1.450473	0.084762
H_6	-2.666222	0.577695	0.130250
H_7	-2.023547	-1.175286	0.024654

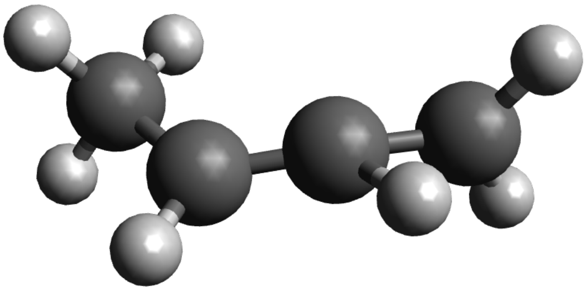

Figure S31. Structure diagram for the transition state **M3-4** optimized at the G4 level of theory

Table S26. Atom coordinates for the transition state **M3-4** in angstroms (Å) optimized at the G4 level of theory

Atom Type	X Coordinate (Å)	Y Coordinate (Å)	Z Coordinate (Å)
C_1	1.764452	0.394739	-0.000109
C_2	0.615874	-0.562604	0.000295
C_3	-0.628940	-0.187492	-0.000355
C_4	-1.875720	0.319308	0.000147
H_1	2.379820	0.203508	0.879210
H_2	1.443118	1.431979	-0.000451
H_3	2.379775	0.202989	-0.879340
H_4	0.831035	-1.625858	0.000882
H_5	-2.405065	0.480330	0.937646
H_6	-2.406059	0.479136	-0.936988
H_7	-1.476618	-0.955790	-0.000823

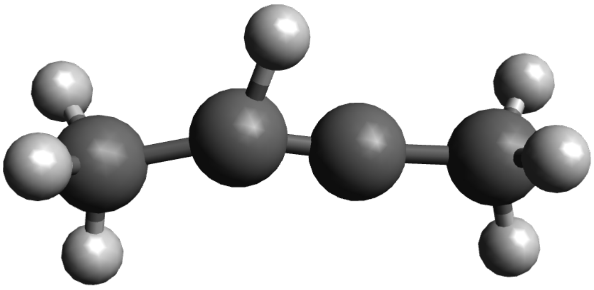

Figure S32. Structure diagram for the transition state **M3-6** optimized at the G4 level of theory

Table S27. Atom coordinates for the transition state **M3-6** in angstroms (Å) optimized at the G4 level of theory

Atom Type	X Coordinate (Å)	Y Coordinate (Å)	Z Coordinate (Å)
C_1	2.052018	0.116002	0.000002
C_2	0.603969	-0.147219	0.000023
C_3	-0.622380	0.006356	-0.000032
C_4	-2.057775	0.071575	-0.000002
H_1	2.508635	-0.317069	0.887667
H_2	2.201787	1.193406	0.000004
H_3	2.508578	-0.317035	-0.887716
H_4	0.180568	-1.247307	-0.000027
H_5	-2.294386	1.142772	0.000509
H_6	-2.480103	-0.367122	-0.902851
H_7	-2.480072	-0.367926	0.902469

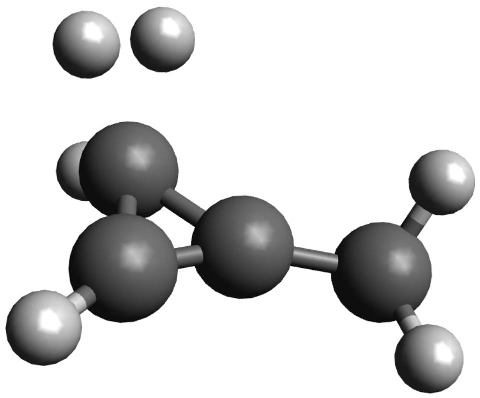

Figure S33. Structure diagram for the transition state **M3-W3** optimized at the G4 level of theory

Table S28. Atom coordinates for the transition state **M3-W3** in angstroms (Å) optimized at the G4 level of theory

Atom Type	X Coordinate (Å)	Y Coordinate (Å)	Z Coordinate (Å)
C_1	-0.490093	-1.577234	-0.025379
C_2	-0.234876	-0.154640	0.250346
C_3	-0.251490	0.926906	0.965438
C_4	0.212088	1.081127	-0.398496
H_1	-1.142165	-1.673442	-0.896946
H_2	-0.954507	-2.052224	0.837909
H_3	0.452315	-2.082661	-0.255117
H_4	1.521718	0.844388	-0.765186
H_5	-0.485897	1.495749	1.850803
H_6	1.513364	1.541658	-0.312411
H_7	-0.140456	1.650372	-1.250960

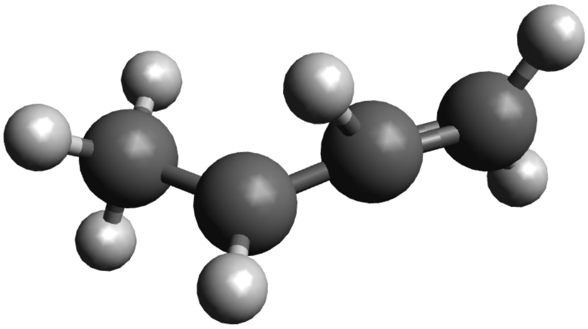

Figure S34. Structure diagram for the transition state **M4-5** optimized at the G4 level of theory

Table S29. Atom coordinates for the transition state **M4-5** in angstroms (Å) optimized at the G4 level of theory

Atom Type	X Coordinate (Å)	Y Coordinate (Å)	Z Coordinate (Å)
C_1	1.750046	-0.387221	0.040992
C_2	0.594568	0.521146	-0.135432
C_3	-0.709417	0.170909	0.018611
C_4	-1.938804	-0.237863	-0.031332
H_1	1.463707	-1.375307	0.386796
H_2	2.507212	0.059872	0.683196
H_3	2.196902	-0.470533	-0.956884
H_4	-0.217380	0.636613	1.043645
H_5	-2.328413	-0.608244	-0.972222
H_6	-2.589855	-0.211786	0.831001
H_7	0.789471	1.567555	-0.372568

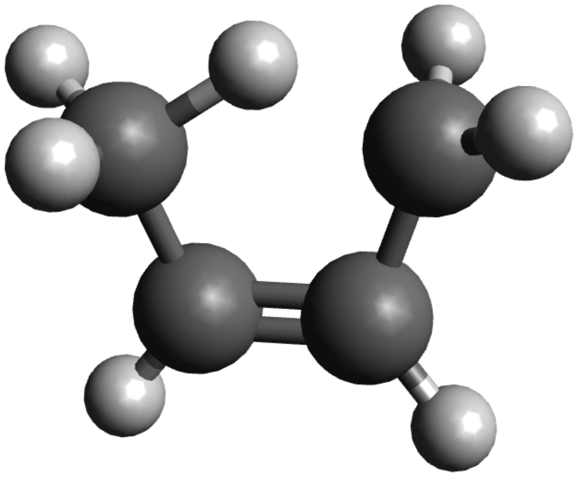

Figure S35. Structure diagram for the transition state **M4-7** optimized at the G4 level of theory

Table S30. Atom coordinates for the transition state **M4-7** in angstroms (Å) optimized at the G4 level of theory

Atom Type	X Coordinate (Å)	Y Coordinate (Å)	Z Coordinate (Å)
C_1	1.362099	-0.351036	0.077924
C_2	0.642897	0.864138	-0.213812
C_3	-0.683435	0.693533	0.024106
C_4	-1.036016	-0.689963	-0.001217
H_1	0.303787	-1.112916	0.098578
H_2	1.487143	-0.579541	1.137059
H_3	2.214902	-0.653738	-0.524000
H_4	1.126295	1.766684	-0.564226
H_5	-1.405428	1.469521	0.244434
H_6	-1.789508	-1.096093	0.669704
H_7	-0.964587	-1.219583	-0.949401

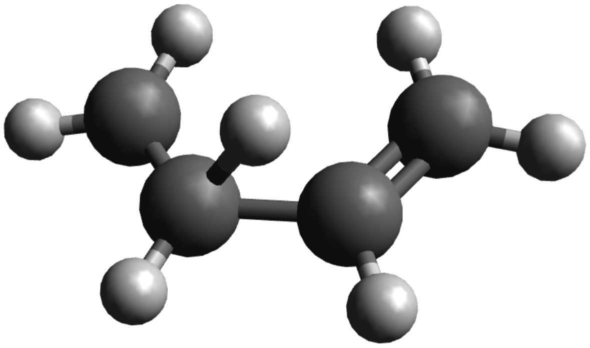

Figure S36. Structure diagram for the transition state **M4-4** optimized at the G4 level of theory

Table S31. Atom coordinates for the transition state **M4-4** in angstroms (Å) optimized at the G4 level of theory

Atom Type	X Coordinate (Å)	Y Coordinate (Å)	Z Coordinate (Å)
C_1	1.862523	-0.123248	0.002624
C_2	0.637410	0.433886	-0.056706
C_3	-0.649660	-0.437824	-0.066625
C_4	-0.577658	-1.772940	-0.042473
H_1	2.001814	-1.177051	0.212823
H_2	0.067730	0.035598	0.968151
H_3	2.747847	0.476932	-0.160233
H_4	0.486571	1.492575	-0.215743
H_5	-1.581212	0.110095	-0.039466
H_6	-1.474150	-2.363490	0.087700
H_7	0.363324	-2.301449	-0.132011

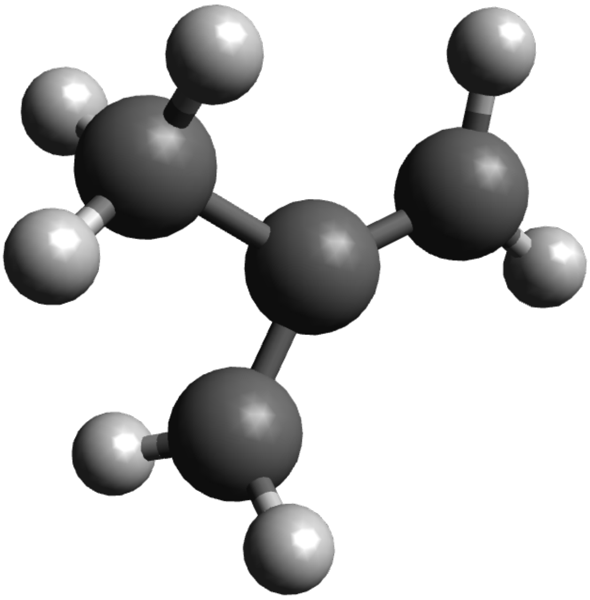

Figure S37. Structure diagram for the transition state **M5-8** optimized at the G4 level of theory

Table S32. Atom coordinates for the transition state **M5-8** in angstroms (Å) optimized at the G4 level of theory

Atom Type	X Coordinate (Å)	Y Coordinate (Å)	Z Coordinate (Å)
C_1	0.765681	1.047202	0.000000
C_2	-0.315539	0.204118	0.000000
C_3	-1.592171	-0.057038	0.000000
C_4	0.893899	-0.980996	0.000000
H_1	1.320442	1.242241	-0.914136
H_2	1.129100	1.487629	0.924876
H_3	-1.969011	-1.069184	0.000000
H_4	-2.299734	0.761065	0.000000
H_5	0.675775	-1.496023	-0.929485
H_6	1.954918	-0.708559	0.000000
H_7	0.675775	-1.496023	0.929485

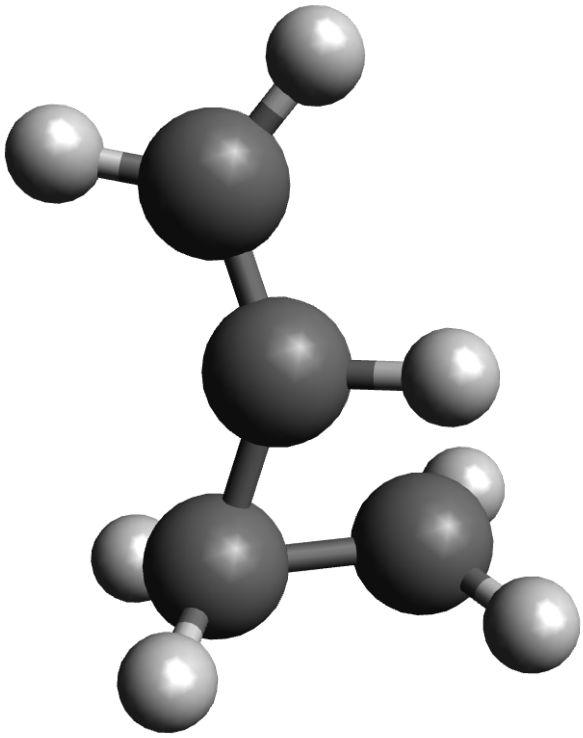

Figure S38. Structure diagram for the transition state **M5-9** optimized at the G4 level of theory

Table S33. Atom coordinates for the transition state **M5-9** in angstroms (Å) optimized at the G4 level of theory

Atom Type	X Coordinate (Å)	Y Coordinate (Å)	Z Coordinate (Å)
C_1	1.209483	-0.622040	0.000970
C_2	0.807726	0.817552	-0.000068
C_3	-0.394751	-0.013204	-0.001298
C_4	-1.708270	-0.069257	0.000435
H_1	1.528149	-1.082298	-0.928033
H_2	-0.040066	-1.110562	-0.005513
H_3	1.522573	-1.082629	0.931667
H_4	1.010755	1.365652	0.911389
H_5	-2.261697	-0.996860	-0.000063
H_6	-2.258304	0.863365	0.001619
H_7	1.013469	1.365024	-0.911303

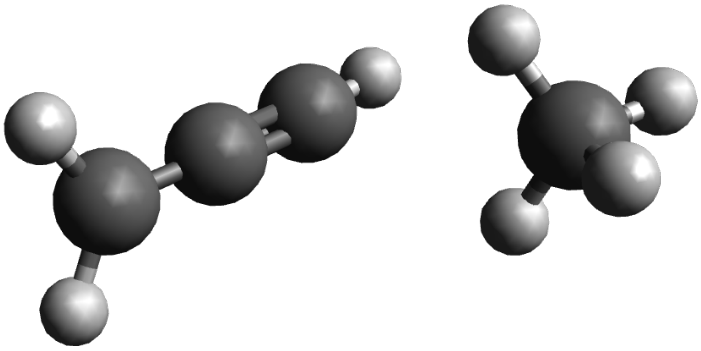

Figure S39. Structure diagram for the transition state **M5-W4** optimized at the G4 level of theory

Table S34. Atom coordinates for the transition state **M5-W4** in angstroms (Å) optimized at the G4 level of theory

Atom Type	X Coordinate (Å)	Y Coordinate (Å)	Z Coordinate (Å)
C_1	0.039644	1.033307	-0.000011
C_2	1.003269	0.262736	0.000000
C_3	2.071959	-0.538644	0.000001
C_4	-2.300777	-0.457184	0.000003
H_1	2.522225	-0.871222	0.935701
H_2	2.522048	-0.871462	-0.935698
H_3	-0.703746	1.804601	-0.000058
H_4	-1.762057	-0.200564	0.922655
H_5	-2.448059	-1.540379	0.000144
H_6	-3.252567	0.078440	0.000171
H_7	-1.762412	-0.200704	-0.922877

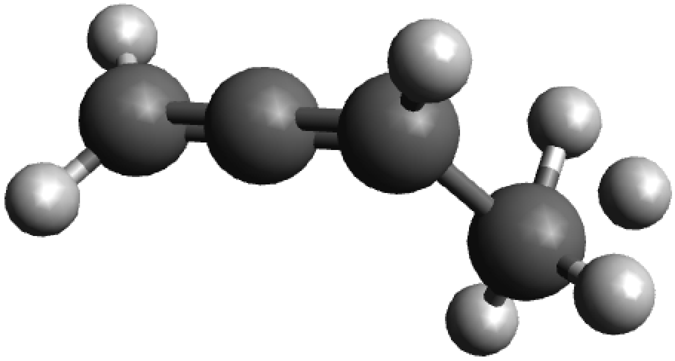

Figure S40. Structure diagram for the transition state **M5-W5** optimized at the G4 level of theory

Table S35. Atom coordinates for the transition state **M5-W5** in angstroms (Å) optimized at the G4 level of theory

Atom Type	X Coordinate (Å)	Y Coordinate (Å)	Z Coordinate (Å)
C_1	-1.3764288182	-0.2396004545	-0.2052170909
C_2	-0.1977338182	0.7289625455	-0.0681050909
C_3	1.0334151818	0.2799855455	-0.0493410909
C_4	2.2590781818	-0.1354194545	-0.0588170909
H_1	-2.2311378182	0.1980805455	-0.7274780909
H_2	-2.1340758182	-0.5952694545	0.7656929091
H_3	-1.3923288182	-0.1515534545	1.0212319091
H_4	-1.1002838182	-1.2444744545	-0.5213740909
H_5	-0.4493248182	1.7831635455	-0.0285350909
H_6	2.7927211818	-0.2902554545	-0.9935250909
H_7	2.7960991818	-0.3336194545	0.8654679091

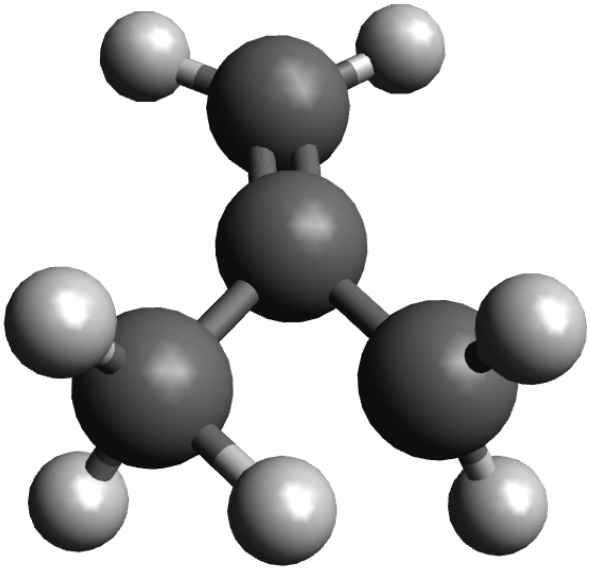

Figure S41. Structure diagram for the transition state **M8-8** optimized at the G4 level of theory

Table S36. Atom coordinates for the transition state **M8-8** in angstroms (Å) optimized at the G4 level of theory

Atom Type	X Coordinate (Å)	Y Coordinate (Å)	Z Coordinate (Å)
C_1	-1.589201	-0.348780	0.000872
C_2	-0.300592	-0.120604	-0.000055
C_3	0.988861	-0.842855	0.001770
C_4	0.691815	0.934157	-0.002714
H_1	-2.296125	0.468246	-0.001131
H_2	-1.974071	-1.358220	0.003418
H_3	1.305771	-1.300416	-0.931559
H_4	1.305325	-1.296148	0.937339
H_5	0.939075	1.438757	0.927847
H_6	1.783656	0.116653	-0.000149
H_7	0.939247	1.433616	-0.936007

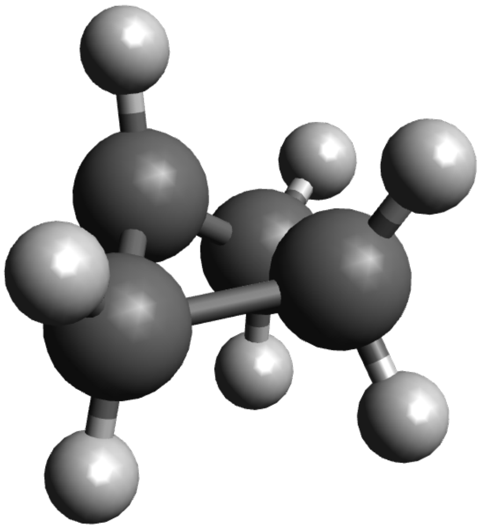

Figure S42. Structure diagram for the transition state **M9-9** optimized at the G4 level of theory

Table S37. Atom coordinates for the transition state **M9-9** in angstroms (Å) optimized at the G4 level of theory

Atom Type	X Coordinate (Å)	Y Coordinate (Å)	Z Coordinate (Å)
C_1	-1.188915	-0.241265	0.2619215
C_2	-0.000058	-0.881639	-0.199996
C_3	0.000128	0.791249	-0.276436
C_4	1.188883	-0.241318	0.261942
H_1	-2.090017	-0.348644	-0.331619
H_2	-1.334612	-0.042480	1.318565
H_3	-0.000084	-1.337291	-1.188336
H_4	-0.000078	1.573483	0.474383
H_5	0.000147	1.119469	-1.307449
H_6	1.334637	-0.042688	1.318609
H_7	2.089968	-0.348881	-0.331589

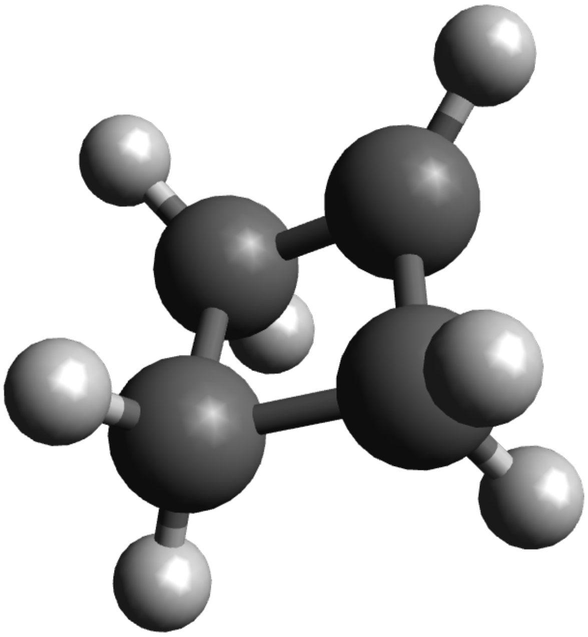

Figure S43. Structure diagram for the transition state **M9-10** optimized at the G4 level of theory

Table S38. Atom coordinates for the transition state **M9-10** in angstroms (Å) optimized at the G4 level of theory

Atom Type	X Coordinate (Å)	Y Coordinate (Å)	Z Coordinate (Å)
C_1	0.020162	1.005875	-0.174980
C_2	1.158020	-0.084768	0.148040
C_3	0.002246	-0.881812	-0.181251
C_4	-1.115340	-0.077126	0.134234
H_1	-0.066303	1.760298	0.595960
H_2	0.002726	1.422272	-1.173395
H_3	1.417649	-0.044957	1.208812
H_4	2.047653	-0.176125	-0.469816
H_5	-1.388438	-0.008113	1.190148
H_6	-1.994737	-0.181845	-0.496072
H_7	-0.085604	-1.724931	-0.860672

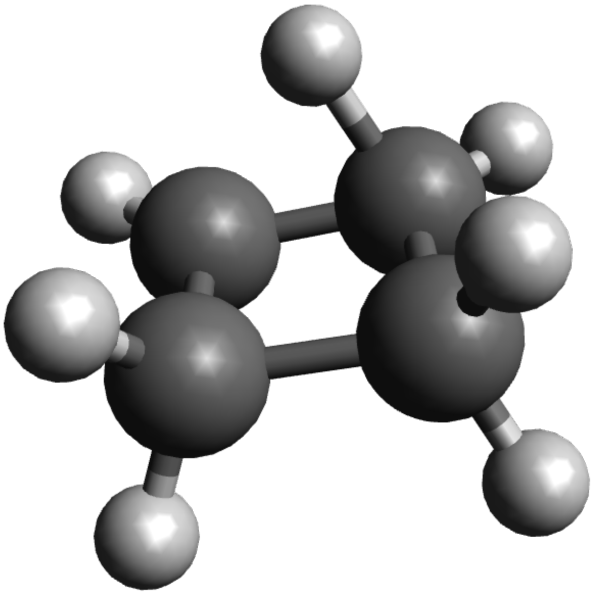

Figure S44. Structure diagram for the transition state **M10-11** optimized at the G4 level of theory

Table S39. Atom coordinates for the transition state **M10-11** in angstroms (Å) optimized at the G4 level of theory

Atom Type	X Coordinate (Å)	Y Coordinate (Å)	Z Coordinate (Å)
C_1	-0.000142	-1.058450	-0.084978
C_2	-1.068064	0.064502	0.057320
C_3	0.000142	1.033245	-0.064888
C_4	1.068073	0.064253	0.057258
H_1	-0.000145	-1.832157	0.673342
H_2	-0.000246	-1.500293	-1.076327
H_3	-0.598121	0.737675	1.164808
H_4	-1.920016	0.178494	-0.618733
H_5	1.494981	0.128549	1.073464
H_6	1.920356	0.178109	-0.618403
H_7	0.000236	2.097036	-0.294999

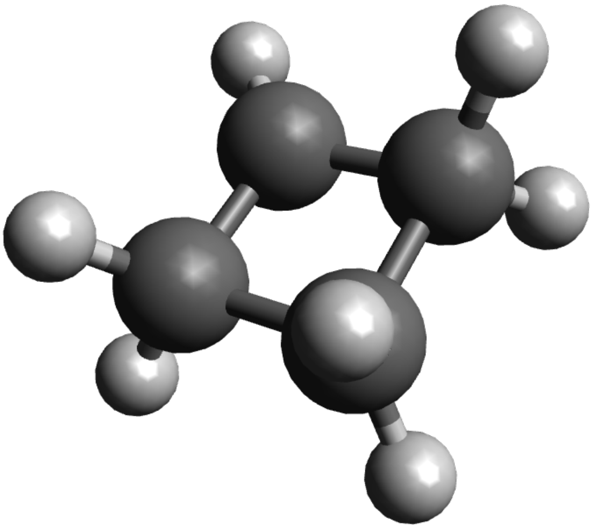

Figure S45. Structure diagram for the transition state **M10-10** optimized at the G4 level of theory

Table S40. Atom coordinates for the transition state **M10-10** in angstroms (Å) optimized at the G4 level of theory

Atom Type	X Coordinate (Å)	Y Coordinate (Å)	Z Coordinate (Å)
C_1	0.000000	0.000000	-1.049186
C_2	0.000000	-1.091240	0.041842
C_3	0.000000	0.000000	1.133211
C_4	0.000000	1.091240	0.041842
H_1	0.906333	0.000000	-1.689902
H_2	-0.906333	0.000000	-1.689902
H_3	0.906794	-1.731543	0.041783
H_4	-0.906794	-1.731543	0.041783
H_5	0.906794	1.731543	0.041783
H_6	-0.906794	1.731543	0.041783
H_7	0.000000	0.000000	2.203211

1. Gardiner, S.H.; Karsili, T.N.V.; Lipciuc, M.L.; Wilman, E.; Ashfold, M.N.R.; Vallance, C. Fragmentation dynamics of the ethyl bromide and ethyl iodide cations: a velocity-map imaging study. *Phys. Chem. Chem. Phys.* **2014**, *16*, 2167–2178. https://doi.org/10.1039/C3CP53970A.
2. Tsai, B.P.; Werner, A.S.; Baer, T. A photoion–photoelectron coincidence (PIPECO) study of fragmentation rates and kinetic energy release in energy selected metastable ions. *J. Chem. Phys.* **1975**, *63*, 4384–4392. https://doi.org/10.1063/1.431155.
3. Booze, J.A.; Weitzel, K.M.; Baer, T. The rates of HCl loss from energy-selected ethylchloride ions: A case of tunneling through an H-atom transfer barrier. *J. Chem. Phys.* **1991**, *94*, 3649–3656. https://doi.org/10.1063/1.459736.
4. Linstrom, P.J.; Mallard, W.G. NIST Chemistry WebBook - Standard Reference Database n. 69. [Online], accessed May 2023. https://doi.org/10.18434/T4D303.
5. Ruscic, B.; Bross, D. Active Thermochemical Tables (ATcT) values based on ver. 1.130 of the Thermochemical Network. [Online], 2022. https://doi.org/10.17038/CSE/1997229.
6. Kuck, D. Half a century of scrambling in organic ions: Complete, incomplete, progressive and composite atom interchange. *Int. J. Mass Spectrom.* **2002**, *213*, 101–144. https://doi.org/10.1016/S1387-3806(01)00533-4.
