# Supplementary figures and images for: Reactivity of the Ethenium Cation (C2H5+) with Ethyne (C2H2): A Combined Experimental and Theoretical Study
Source: Molecules. 2024 Feb 9;29(4):810. doi: 10.3390/molecules29040810 (PMC10892252; doi:10.3390/molecules29040810)

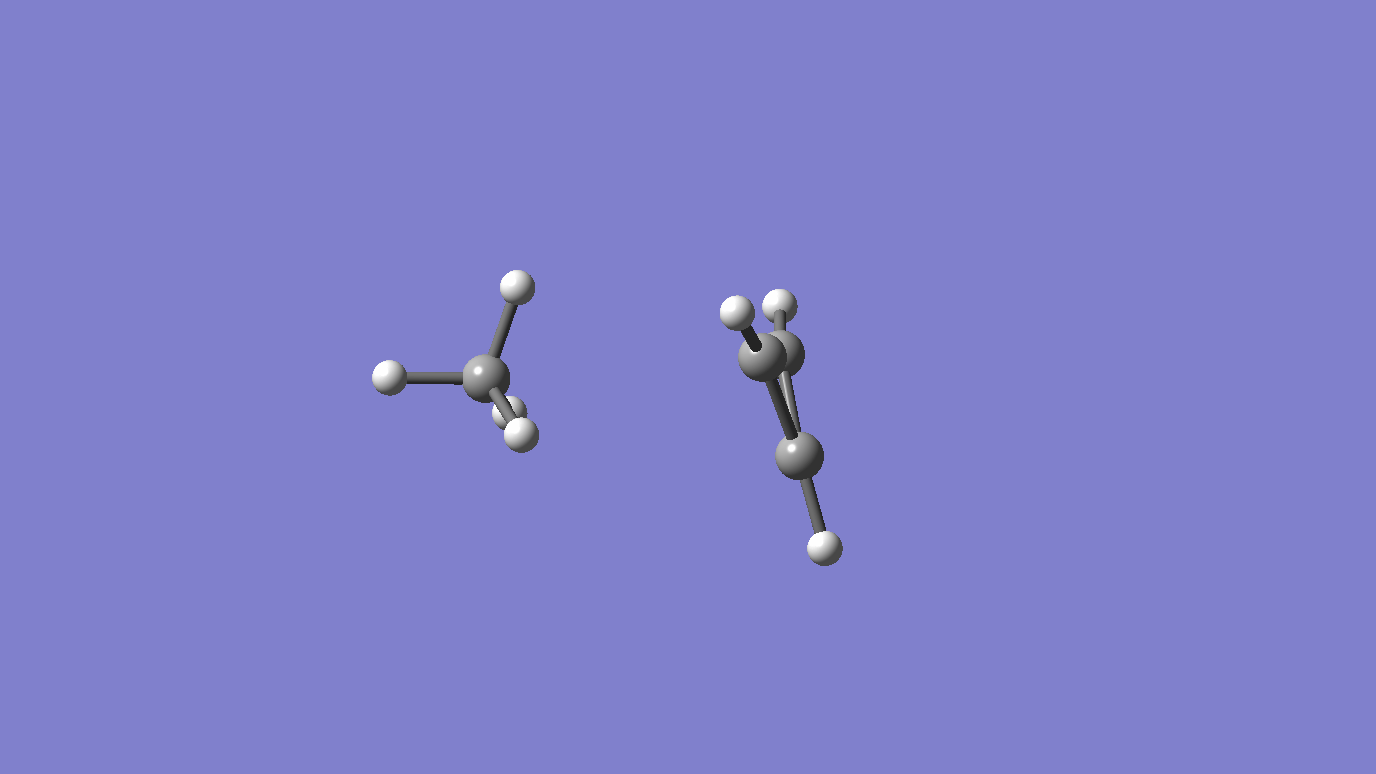

Supplement: Supplementary file 1 [file molecules-29-00810-s001.zip › TS M1-W2.gif]

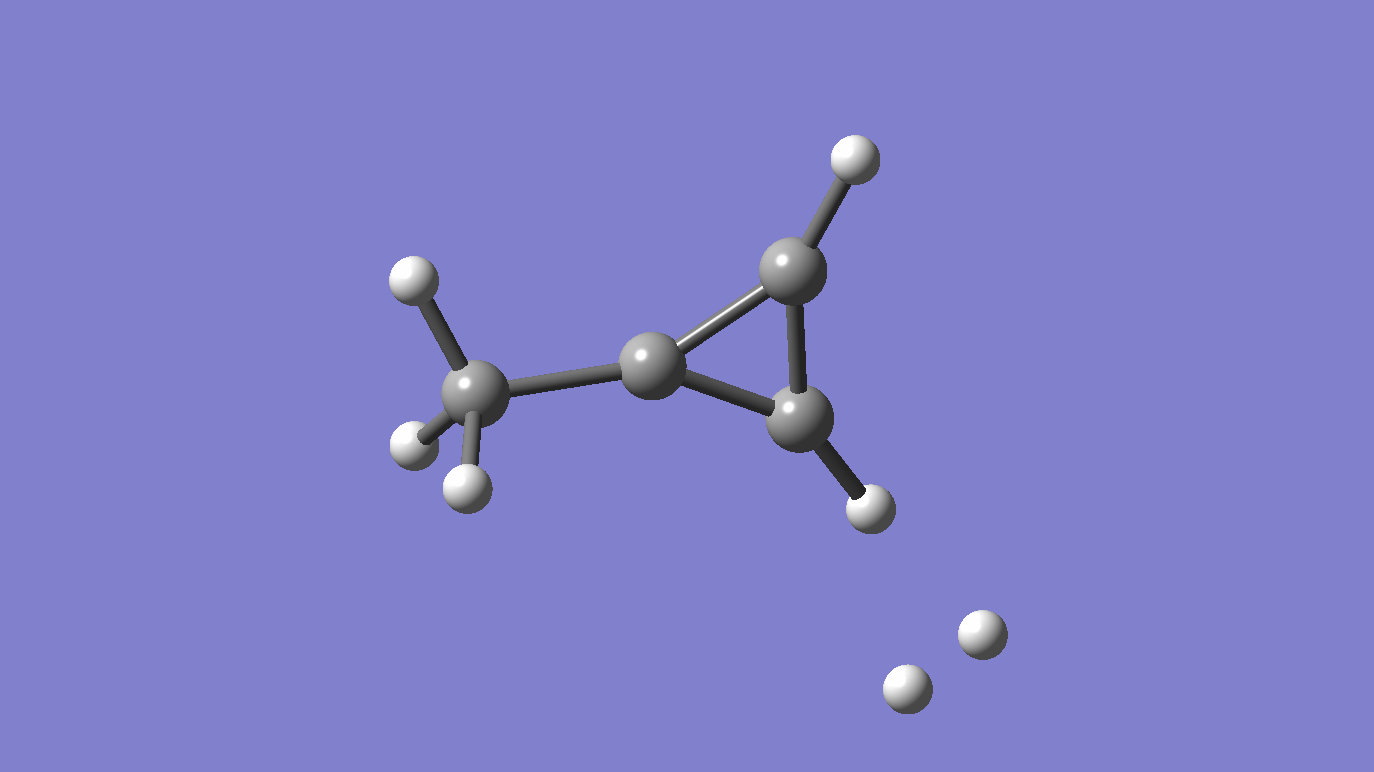

Supplement: Supplementary file 1 [file molecules-29-00810-s001.zip › TS M3-W3.gif]

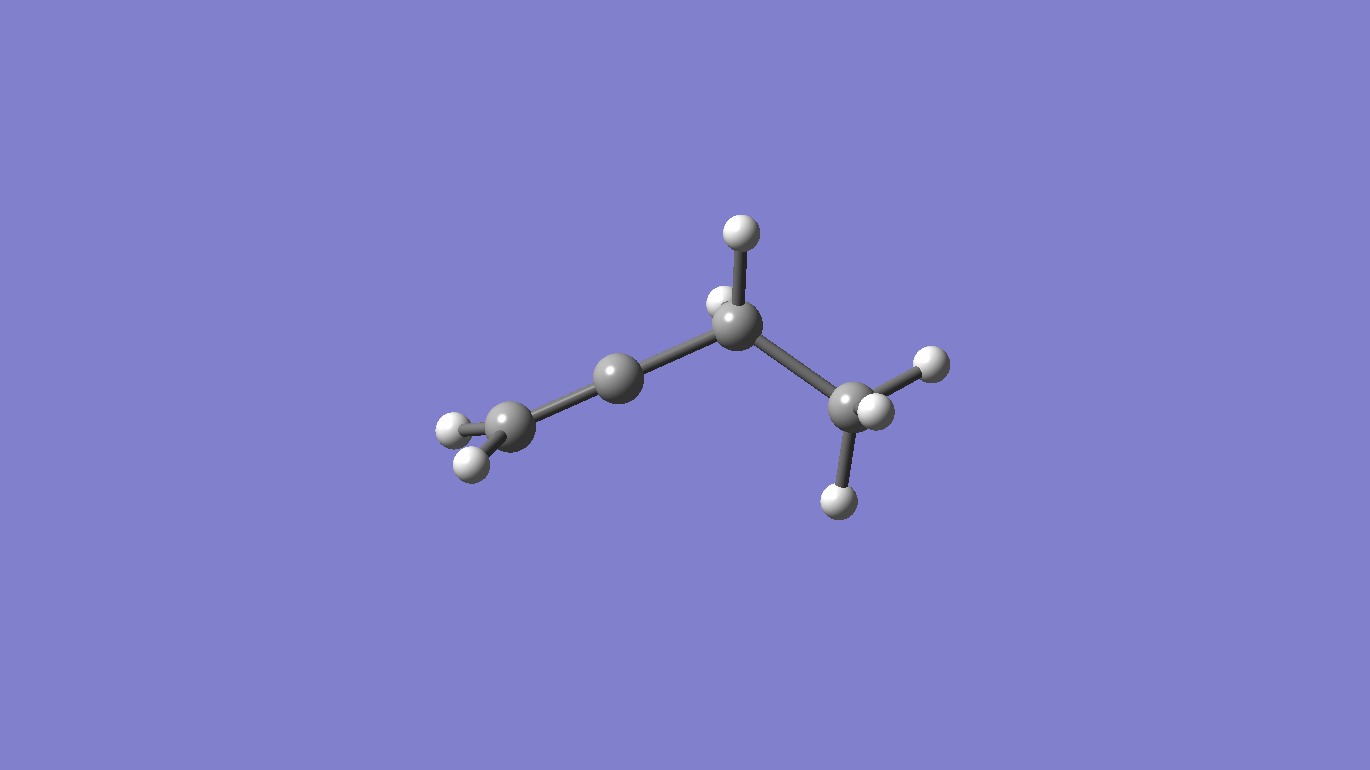

Supplement: Supplementary file 1 [file molecules-29-00810-s001.zip › TS M5-W4.gif]

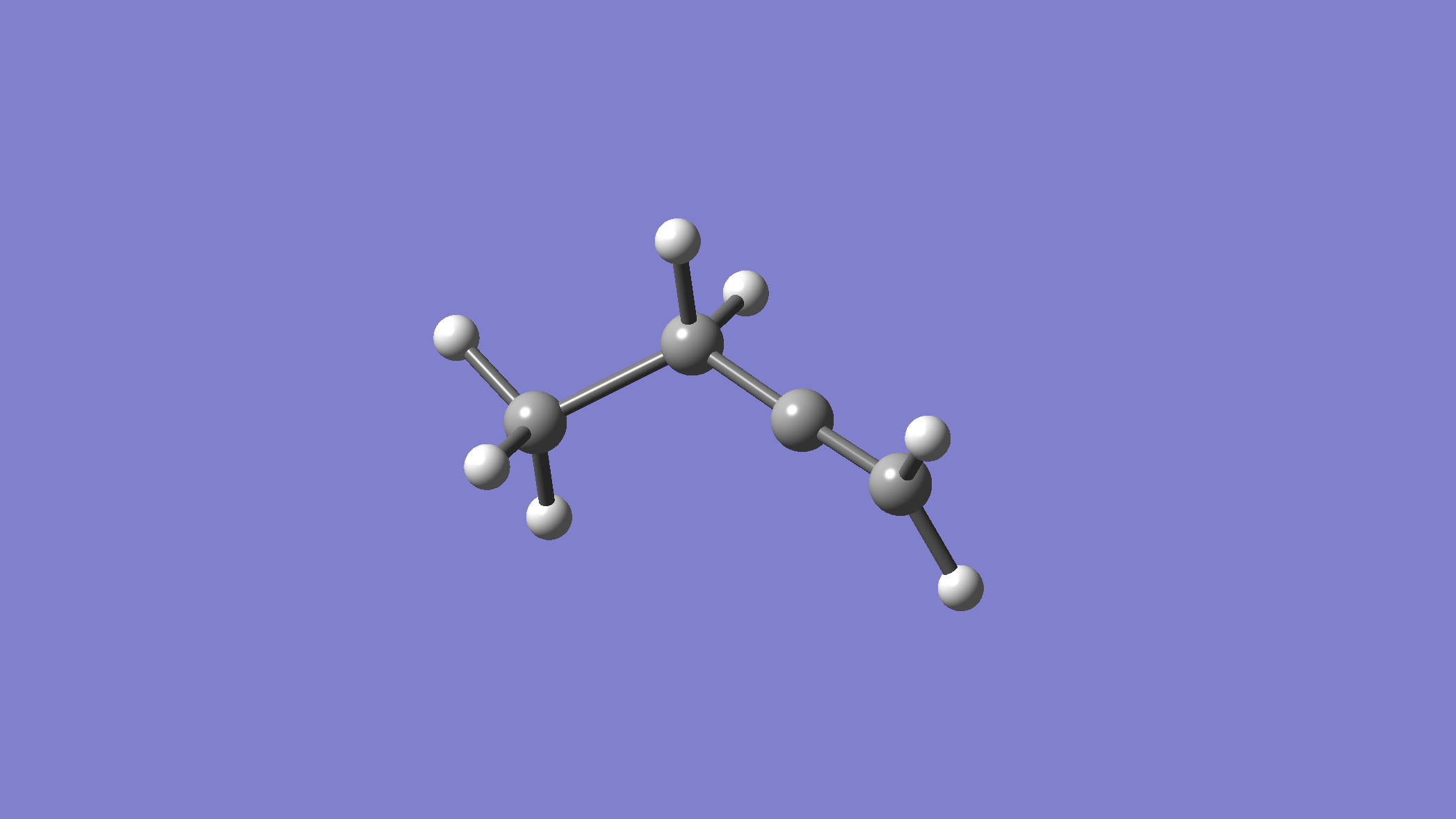

Supplement: Supplementary file 1 [file molecules-29-00810-s001.zip › TS M5-W5.gif]
